# Porous metal implants: processing, properties, and challenges

**DOI:** 10.1088/2631-7990/acdd35

**Published:** 2023-07-13

**Authors:** Amit Bandyopadhyay, Indranath Mitra, Jose D Avila, Mahadev Upadhyayula, Susmita Bose

**Affiliations:** 1 W. M. Keck Biomedical Materials Research Lab, School of Mechanical and Materials Engineering, Washington State University, Pullman, WA 99164, United States of America

**Keywords:** porous metals, load-bearing implants, 3d printing, additive manufacturing, mechanical properties, biological properties

## Abstract

Porous and functionally graded materials have seen extensive applications in modern biomedical devices—allowing for improved site-specific performance; their appreciable mechanical, corrosive, and biocompatible properties are highly sought after for lightweight and high-strength load-bearing orthopedic and dental implants. Examples of such porous materials are metals, ceramics, and polymers. Although, easy to manufacture and lightweight, porous polymers do not inherently exhibit the required mechanical strength for hard tissue repair or replacement. Alternatively, porous ceramics are brittle and do not possess the required fatigue resistance. On the other hand, porous biocompatible metals have shown tailorable strength, fatigue resistance, and toughness. Thereby, a significant interest in investigating the manufacturing challenges of porous metals has taken place in recent years. Past research has shown that once the advantages of porous metallic structures in the orthopedic implant industry have been realized, their biological and biomechanical compatibility—with the host bone—has been followed up with extensive methodical research. Various manufacturing methods for porous or functionally graded metals are discussed and compared in this review, specifically, how the manufacturing process influences microstructure, graded composition, porosity, biocompatibility, and mechanical properties. Most of the studies discussed in this review are related to porous structures for bone implant applications; however, the understanding of these investigations may also be extended to other devices beyond the biomedical field.

## Highlights

Porous metals are extensively used in load-bearing implants to improve osseointegration.
Different processing approaches for porous metals are discussed here.
Static and dynamic mechanical properties are critically reviewed for porous metal implants.
*In vitro* and *in vivo* biological properties of porous metal implants are critically reviewed.
Current challenges and future directions for porous metal implants are discussed.

## 1. Introduction

Porous metals were first introduced in 77 AD when goldsmiths used a newly discovered granulation process to prepare aesthetically pleasing jewelry [1]. To this day, derivations of this approach are used when processing porous metals or metallic foams. Modern derivations began in the early 20th century when powderized metals were sintered to form first-of-a-kind porous metals for various engineering applications. Since then, this method has been used successfully to prepare filters, batteries, self-lubricating bearings, etc. [2, 3]. Although the commercial investigation into porous metals began in 1925, the progress was slow until the 1980s [4]. Since then, the widespread commercial availability of varying porous metallic materials for various applications has steadily increased. Porous metals are often the best option in structural engineering, where lightweight and high mechanical properties, high surface area, and/or interconnected porosity are needed. Although porous ceramic [5, 6] and porous polymeric materials [7–11] are also available, porous metallic materials offer unique properties that are often difficult to accomplish with other material systems. For example, porous polymeric materials lack in both sufficient mechanical strength and high-temperature stability. Alternatively, porous ceramic materials possess inherent brittleness, which limits their use in applications requiring high toughness and fatigue resistance [12, 13].

Using porous metals for orthopedic implants began with osseointegration devices [14]. Early on, many disadvantages with the use of dense metals as orthopedic implants were observed; specifically, the main disadvantage was the mismatch in effective Young’s modulus of the bulk implant structure (110 GPa for titanium (Ti); 193 GPa for stainless steel (SS316L); 220–230 GPa for cobalt-chrome alloys) and the surrounding bone, which ranges from 10–30 GPa. Compared to the host tissue, the difference in effective stiffness of the implant inherently allowed for the physical phenomena known as *stress shielding*. When the effective stiffness of the implant is significantly greater than that of the surrounding hard tissue, the implant shields the host tissue from applied loads; this promotes uneven localized loading events and limits bone compliance during mechanical loading. The stress shielding event may cause premature failure by aseptic loosening, where the host tissue becomes idle from insufficient use, also known as *bone resorption*. The second is the separation and fracture at the host tissue–implant interface due to insufficient tissue anchorage [15]; bulk non-porous implant materials tend to exhibit weaker interfacial bonding with the surrounding bone tissue. Surface texturing, such as surface roughening or lattice-designed surface porosity, may remedy this issue and thus lead us to the topic at hand.

The typical lifetime for total hip replacement (THR) remained nearly unchanged for the last fifty years, averaging 10–15 years of operational use [16]. This was particularly so because most THR surgical procedures were performed in patients age 65 or greater, thus, creating a lack of impetus for an implant of a greater operational lifetime. However, a general trend of these procedures being performed on much younger patients has been observed in recent years. With improving medical care and medication, patient self-heath awareness, and the ability to catch early-stage illnesses, the average lifespan of society has increased, and demand for an increase in the operational life of load-bearing articulating implants has also increased. Alternatively, for the younger members of society, greater trust in medical care has allowed for more reparative surgeries, which return normal biomechanical motion of a limb or joint. One challenge associated with implantation into a more youthful society is that the implants will most commonly undergo more significant mechanical stresses with an increased exposure rate. Therefore, a push for improved implant performance and lifespan is imperative; investigating and innovating porous metallic implant structures with stiffness closer to bone and interconnected porosity is deemed highly significant. These implants should promote metabolite and nutrient exchange and allow bone ingrowth leading to improved implant-tissue anchorage and interfacial strength [16]. Furthermore, a porous implant surface allows for the introduction of a compliant layer; this layer should mimic the natural bone joint, such as the hip joint, and reduce wear-related issues [17]. The consolidation of these requirements when considering implant material design will allow for the reduction in revisional surgeries and increase operational life. A significant reason behind the lack of interest in porous metallic materials until recently was the lack of substantial mechanical strength and lower fatigue resistance [13]. However, recent manufacturing techniques have improved the properties of porous metals, and the drive to develop improved and longer-lasting implant structures have reinvigorated interest in the area.

Mechanical properties and the applicability of porous structures for load-bearing implantation sites depend highly on factors such as pore interconnectivity, open porosity, pore size, phases present, compositional variations, grain size, etc. Naturally, most studies investigating porous metal applicability for biomedical implants. Therefore, studying a novel fabrication technique focus on and evaluate the mechanical properties, corrosion resistance, fatigue resistance, and biological compatibility, of biomaterials; all of which are directly linked to the cell morphology and factors of which have an influence on cell attachment and integration into the host tissue.

Material structure cell morphologies, to an extent, tend to depend on the fabrication techniques and, in some cases, the parameters used in the fabrication process. For instance, random foaming processes form closed-cell porosity, where the pore morphology depends on the fabrication process parameters. Similarly, open-cell porous structures may be fabricated by solid-state fusion of metal powder, fibers, decomposition of foaming agents in liquid metals, or vapor deposition. While the solid-state fusion of powdered metals and the vapor deposition methods can produce controlled and higher open-cell porosities, using foaming agents in molten metal matrices usually produces lower and less predictable porosities in closed-cell [18]. Thus, some of the fabrication techniques, based on the type of pore morphology produced, are discussed and tabulated in table 1 [13], and some of the commercially available fully porous and porous-coated implants, their applications and description are reported in table 2 [19].

**Table 1.** Porous metal fabrication techniques categorized based on the pore morphology.

Porosity type	Fabrication methods	Pore distribution
Closed-Cell	Gas Foaming Method	Random
	Decomposition of Foaming agents	
	Plasma Spraying Method	Graded
Open-Cell	Metal powder sintering	Non-homogeneous
	Metal fiber sintering	
	Plasma-spraying	
	Replication	
	Combustion synthesis	
	Space-holder technique	
	Wire mesh oriented orderly	Homogeneous
	Vapor deposition	
	Additive manufacturing	

**Table 2.** Commercially available porous metal structures or porous coatings and their applications.

Fabrication methods	Commercial name	Description	Application
Sintering	CSTi^TM^	Powder Sintering with pressure.	Hip and Knee Surgeries
		Porous Ti coating	
		Porosity: 50%–60%	
		Pore Size: 400 *μ*m–600 *μ*m	
		Stiffness: 106–115 GPa	
	CoCr beads	Powder Sintering	Hip Surgery
		Porous CoCrMo coating	
		Porosity: 30%–50%	
		Pore Size: 100 *μ*m–400 *μ*m	
		Stiffness: 206 GPa	
	Fiber Metal	Sintering	
		Porous Ti coating	
		Porosity: 40%–50%	
		Pore Size: 100 *μ*m–400 *μ*m	
		Stiffness: 106–115 GPa	
Vapor Deposition	Tritanium^TM^	Low-temperature arc vapor deposition of the polyurethane foam shell	
		Porous Ti coating	
		Porosity: 60%	
		Pore Size: 616 *μ*m	
		Stiffness: 106–115 GPa	
		Chemical Vapor Deposition infiltrating Carbon skeleton.	
	Trabecular Metal^TM^	Open cell	Hip and Knee Surgeries
		Porous Ta	
		Porosity: 75%–85%	
		Pore Size: 550 *μ*m	
		Stiffness: 2.5–3.9 GPa	
		Porous Ti	
Porous Plasma Processing	Regenerex^TM^	Porosity: 67%	Hip and Shoulder Surgeries
		Pore Size: 300 *μ*m	
		Stiffness: 1.6 GPa	
		Porous Ti coating	
	Stiktite^TM^	Porosity: 60%	Hip and Knee Surgeries
		Pore Size: 500 *μ*m	
		Stiffness: 106–115 GPa	

**Figure 1.**
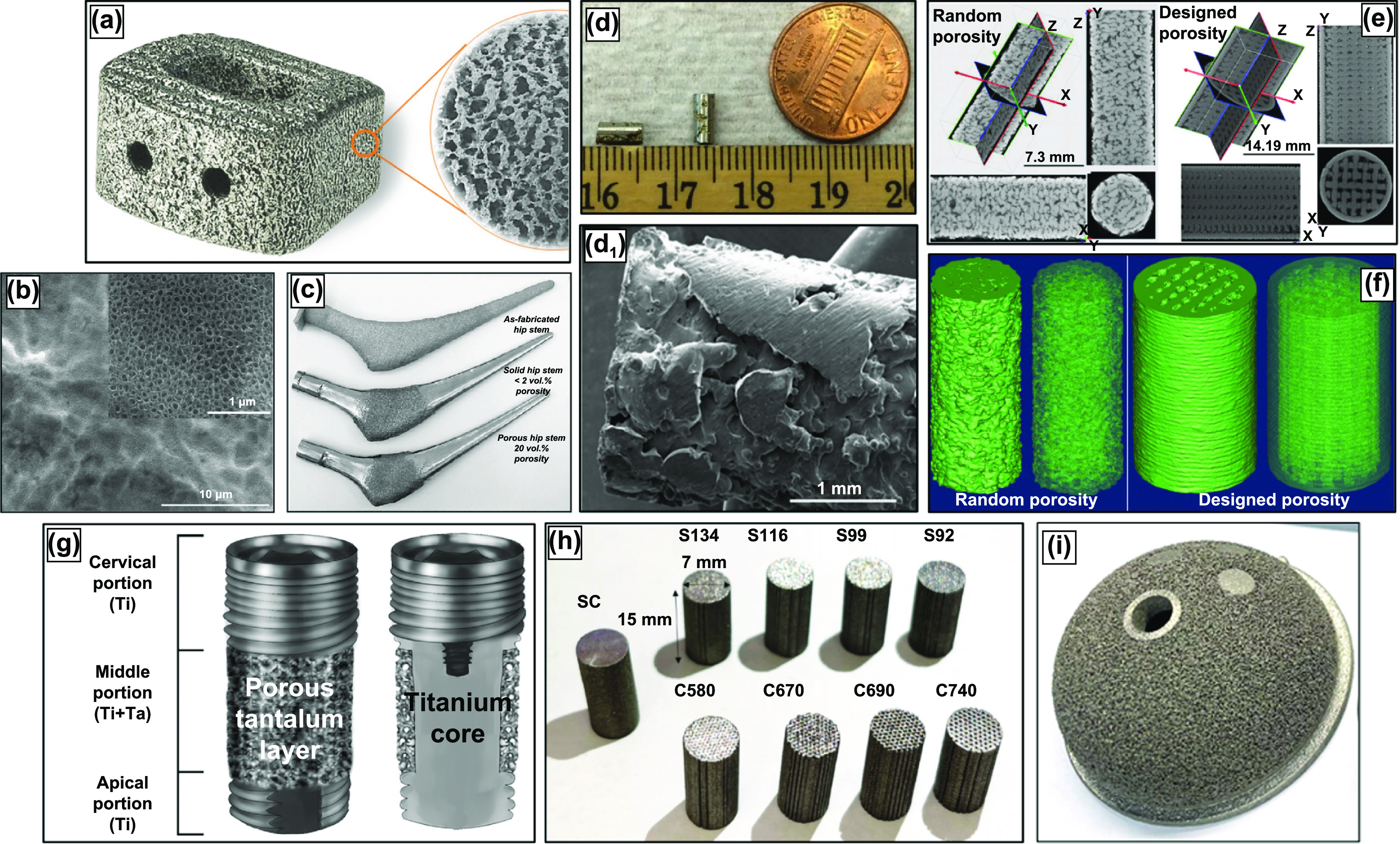
AM-produced porous-metal structures by the research and industrial sector. (a) Porous Nitinol cage for anterior intervertebral fusion produced by combustion synthesis, higher magnification image displaying the irregular pore morphology. Reproduced from [20]. CC BY 4.0. (b) Fabricated TiO_2_ nanotubes on the surface of porous Ti implant. Reproduced from [21], with permission from Springer Nature. (c) Porous hip stems produced with varying porosity. Reprinted from [22], Copyright (2010), with permission from Elsevier. (d) DED-LENS^TM^ *in vivo* cylindrical samples with 25% porosity. Reproduced from [21], with permission from Springer Nature. (e) Micro CT imaging of DED produced porous Ti samples and (f) the respective 3D reconstruction [23], 2010, reprinted by permission of the publisher (Taylor & Francis Ltd, www.tandfonline.com.) (g) Porous tantalum trabecular metal (PTTM) dental implant displaying the Ti body and Ta porous surface [24], John Wiley & Sons. © 2013 Wiley Periodicals, Inc. (h) 3D systems powder bed fusion produced samples with varying cell-size and strut-size. Reprinted from [25], Copyright (2022), with permission from Elsevier and (i) powder bed fusion produced porous acetabular cup.

With each technique, challenges may arise, but porous implants have inherent challenges that need to be resolved. Porous structures display lower mechanical strength, which is disadvantageous for load-bearing and load-bearing articulating applications. The fatigue life of porous structures is significantly reduced due to stress concentration sites at the neck region of each pore. The increased surface area of porous structures tends to increase corrosion rates. Moreover, as mentioned before, surface porosity affects implant anchoring by influencing bone ingrowth. Porous metal structures’ corrosion and wear properties depend on the structure’s porosity, hardness, and resistance to plastic deformation. It is necessary to understand a porous structure’s physical, mechanical, and biological properties based on its porosity, microstructure, and composition, some of which vary drastically depending on the fabrication process. Therefore, in this review article, different fabrication processes for manufacturing porous metal structures have been discussed; microstructural and compositional influences of these processes have been elaborated on. Additionally, an effort has been made to compare porous implant structures’ physical, mechanical, and biological properties based on their fabrication process. In summary, a knowledge-based discussion of the challenges and possible future direction for the fabrication and application of porous metallic implants is presented; some currently produced implants have been summarized in figure 1.

## 2. Fabrication methods for porous metals

Porous metal structures can be classified into closed-cell and open-cell porous structures, as listed in table 1. Considering that the type of porosity is dependent, to an extent, on the fabrication process, several fabrication methods have been discussed in this section.

### 2.1. Fabrication methods for closed-cell porous metals

While closed-cell porous metal structures might be helpful in various industrial applications, their use in orthopedic implants has certain limitations. Closed porosity reduces a porous structure’s effective modulus and density, reducing the susceptibility for the occurrence of stress shielding. However, bone ingrowth would be compromised due to the lack of interconnected open porosity and surface wettability, thus limiting protein adhesion and subsequent cell attachment. Therefore, bone cement or another fixation means must hold the implant for osseointegration. Again, depending on the fabrication method used, the closed-cell pores can be either random or graded in distribution. The method for producing metal foams from molten metal due to gas introduction can be categorized into gas foaming and *in situ* gas generation by decomposition of foaming agents [26]. While gas injection into molten metal and decomposition of foaming agents would produce randomly distributed porosity, plasma spraying would produce graded pore distribution [13].

#### 2.1.1. Gas foaming method.

**Figure 2.**
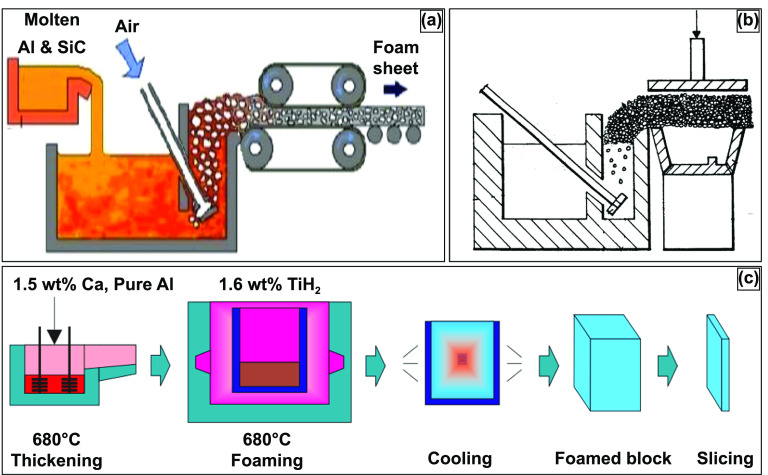
Two established processes for producing metal foam structures. (a) Schematic diagram of the melt gas injected (MGI) process. Reproduced from [35]. CC BY 4.0. (b) Sectional view of an alternate MGI process for manufacturing complex shapes. Reproduced from [34]. CC BY 2.0. (c) Manufacturing process of ALPORAS for metal foam production. [36] John Wiley & Sons. © 2000 WILEY-VCH Verlag GmbH, Weinheim, Fed. Rep. of Germany.

The method of self-formation of metal foams has been considered one of the most economical methods. The porosity in the metal foams generated by such methods is produced via self-evolution based on physical principles. Thereby, the stochastic nature and unpredictability of the cell structure are inevitable. Moreover, since the metal melt has high surface energy and low viscosity, certain additives and surfactants would be required to stabilize the cell walls by lowering surface energy and increasing the viscosity, thereby increasing the bubbles’ stability [19]. A specific method of the gas foaming process is melt gas injection (MGI), as displayed in figure 2; this method involves using conventional foundry procedures to prepare a metal matrix composite consisting of additives and surfactants. In previous work, the additives have been ceramic particles such as SiC or Al_2_O_3_ and are added into the melt. In its molten state, gas is injected as small bubbles throughout the melt; the additives trap the gas bubbles, delay their coalescence due to their higher interfacial energies, and stabilize the cell walls. Moreover, the presence of the additives increases the viscosity, decreasing the velocity of the rising bubbles [26–28]. Then, using a conveyor belt system, the generated foam is carried away, cooled, and solidified. In this process, it was observed that the size of the bubbles was controlled by changing the gas flow rate, the propeller’s design, and the propeller’s speed. The metal foam produced by this method offered a density gradient and elongated or distorted cell morphology with corrugations in the cell wall. Additionally, the produced unit displayed anisotropy, heterogeneity, and lower mechanical properties [29–31]. It was later observed that these metal foam features could be improved by pulling the foam vertically [32]. For more complex part fabrication, numerous attempts were made by casting the semiliquid foam into melds or shaping it using rolls, as displayed in figure 2(b) [33, 34].

#### 2.1.2. Decomposition of foaming agents in a melt.

An alternative method for producing metal foams is by producing gas within the melt itself; this is typically done by adding foaming or blowing agents to the melt and not by gas injection from an external source. The foaming agent decomposes within the melt, generating gas *in situ*, thus producing metal foams [37–39]. Once the desired viscosity is obtained, the melt and the foaming agent are added to the casting mold. The foamed metal expands gradually at a constant pressure conforming to the mold, illustrated in figure 2(c) [40]. The metal foams produced by this technique have displayed a high degree of homogeneity [40, 41]. Typical foaming agents observed in the field have been TiH_2_ and ZrH_2_ [41]. Porous aluminum slabs or cast Al alloys with 10–20 vol. % SiC or Al_2_O_3_ particles have been produced by the decomposition of the foaming agent method [40, 41].

#### 2.1.3. Plasma spraying method.

**Figure 3.**
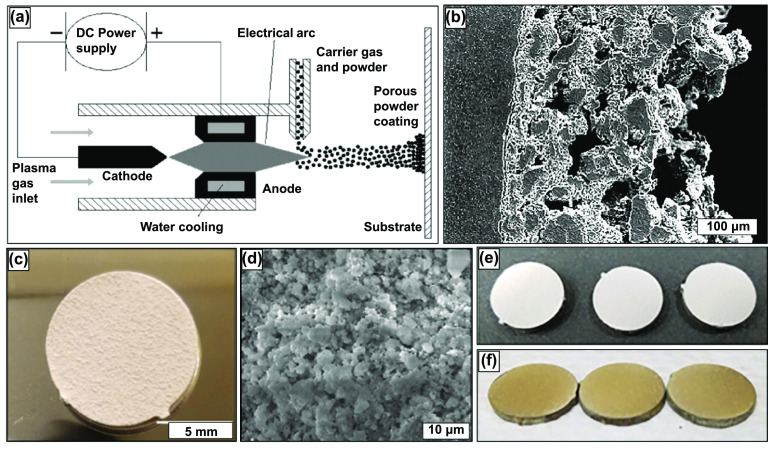
The plasma spray technique along with research produced samples. (a) Schematic representation of plasma spraying process. The porosity of the coating may be varied by altering spraying parameters (b) Cross-sectional view of plasma sprayed coating on a substrate. Reprinted from [13], Copyright (2006), with permission from Elsevier. (c) Induction plasma-sprayed carvacrol/thymol-loaded hydroxyapatite (HA) on Ti64 substrate and (d) high magnification SEM micrographs of after-release study. Reprinted with permission from [46]. Copyright (2020) American Chemical Society. (e) Displays HA-coated and (f) ZnSiAg-HA doped coatings on Ti64 substrate. Reprinted from [47], Copyright (2019), with permission from Elsevier.

Plasma spraying primarily produces functionally graded porous coatings or completely porous parts [42]. Functionally graded porous structures offer a rough and porous surface for the bone tissue to grow within and anchor to the implant. The versatility of this method is such that it can produce porous coatings on dense substrates with porosity >50% [43]. The metal powder is added to the carrier gas, which is injected into the hot plasma jet stream, where the metal powder is brought to its melting point, accelerated, and impinged onto the substrate with high kinetic energy. The plasma spraying process can be done in a controlled environment to avoid the influences of the surrounding atmosphere [42]. The schematic representation of the process is shown in figure 3(a) [13]. The porosity of the coating produced by this method is altered by varying the spray parameters. The porous coating deposited on the substrate comprises of the starting material matrix with some spontaneously generated phases owing to the short reaction time. The porosity of these coatings varied largely in 3 layers: a very dense inner layer that enabled desirable mechanical, metallurgical, and physical bonding properties, a middle layer consisting of a combination of micro and macro pores, and an outer layer that largely contained macro pores [44, 45]. The porosity of these coatings is irregular and has low interconnectivity, figure 3(b) [42], however current research in this field has shown that these coatings can be doped with anti-inflammatory, antibacterial, recovery period and with natural medicinal compounds to promote cell attachment and phenomena such as angiogenesis, as displayed in figures 3(c)–(f) [46, 47]. A. Vu reported that thymol and carvacrol-loaded hydroxyapatite displayed bacterial inhibition of *Staphylococcus epidermidis* and reduced osteoclast resorption pit formation [46]. The objective was to derive a natural medicinal system that would prompt bone healing with antibiotic infection prevention.

### 2.2. Fabrication methods for open-cell porous metals

Most commonly, implants are fixed in place with the help of bone cement or metal screws. However, the major drawback to bone cement is fragmentation which can result in foreign body response to the released debris; this mode of implant fixation usually leads to periprosthetic osteolysis, early aseptic loosening, and failure of the implant [48]. For quicker healing after implantation, osseointegration is critical. Osseointegration, meaning, bone tissue ingrowth into the implant surface. The correlation between the morphology and size of the porous surface and the strength of fixation with the surrounding tissue has also been determined [49]. Many studies have concluded that open porosity improves implant wettability and aids bodily fluids flow, thus improving osseointegration when >100 *μ*m pore size is present [50]. The porosity is either homogeneously or non-homogeneously distributed based on the processing method. Discussed are several fabrication methods for producing open-cell porous implants or coatings.

#### 2.2.1. Sintering.

Sintering is one of the oldest and most evolved techniques in powder metallurgy for producing density-controlled materials. The basic concept of the process is to prepare powder (metal/ceramics) by compacting and binding powdered raw material and providing thermal energy for the compacted powder for densification and grain growth. In this process, the powder particles bond at high temperatures with minor changes to the initial powder particle shape. Binders hold the powder particles together, providing enough area for mass transport during solid-state diffusion. Due to this technique’s highly evolved nature, many studies on porous orthopedic implant structures have employed sintering or modified versions of sintering for preparing porous metal structures.

**Figure 4.**
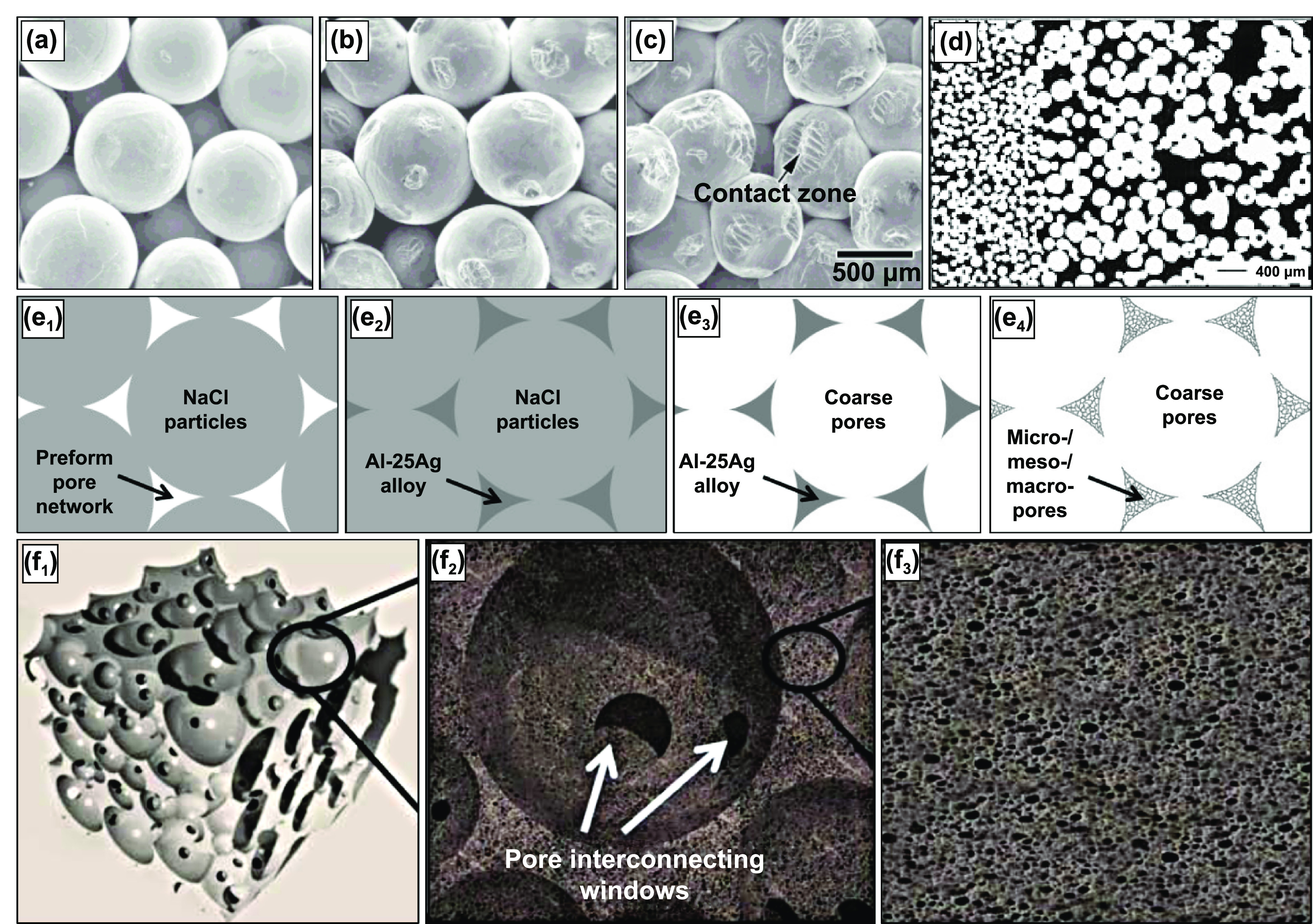
SEM micrographs of the fracture surface microstructure of porous Ti compacts sintered at (a) 1173 K, (b) 1373 K, and (c) 1573 K. Reprinted from [51], Copyright (2003), with permission from Elsevier. Figure (d) displays the cross-sectional microstructure of functionally graded Ti produced by stacked sintering. Reproduced from [45], with permission from Springer Nature. Displayed is a (e) schematic of processing Ag-based foams with hierarchical pore structures: (e_1_) packing NaCl particles (hard template) to obtain a porous preform, (e_2_) infiltrating the porous preform in (e_1_) with Al25Ag, (e_3_) dissolving the NaCl particles in water to generate a foam structure with coarse pores, and (e_4_) selectively dissolving in acidic or alkaline media to generate micro/meso/macropores in the initial foam struts in (e_3_). Various three-dimensional zoom drawing views of hierarchical porous foams produced in the present work: (f_1_) general view, (f_2_) zoomed view showing coarse pores and pore-connecting windows, and (f_3_) zoomed view showing micro/meso/macropores developed in struts. Reprinted with permission from [52]. Copyright (2021) American Chemical Society.

The porosity of the sintered structures can be controlled by tailoring the shape and size of the metal powder, the compaction pressure, and the temperature and time of sintering. It was observed that the compaction pressure and the sintering temperature significantly impact the microstructural and mechanical properties of the porous sintered metals. In general, it was observed that sintered Ti compacts at 1173 K, 1373 K, and 1573 K at no applied pressure; as the temperature increased, the porosity decreased. However, the porosity remained more significant than 30% for each temperature, and almost 100% was observed to be open porosity. Moreover, the effect of different applied pressures at different sintering temperatures has also been studied, and it was observed that the porosity decreases considerably (∼19%). However, most of the porosity still was found to be open porosity. The scanning electron microscope (SEM) images of the microstructure of Ti compacts sintered at 1173 K, 1373 K, and 1573 K with an applied pressure of 10 MPa can be seen in figures 4(a)–(c), respectively [51].

Additional investigations produced porous functionally graded Ti using the sintering method by stacking layers of powders with varying particle sizes and the volume fraction of the additive (silicon), i.e. the low volume fraction of additive with finer powder (20% with 45 *μ*m) to the high-volume fraction of additive with coarser powder (45% with 200 *μ*m). The cross-sectional microstructure of a functionally graded porous Ti structure prepared in this manner is shown in figure 4(d) [45].

Another alternative form of porous surface characteristics to implant the structure’s dense core via sintering is metal fibers instead of metal powder. This method has been investigated for both stainless steel and Ti fibers, and the procedure used to make such porous coatings is similar to the powder metal sintering process [53–56]. The metal fibers are laid complying with the form of the implant structure, compacted, and then sintered at high temperatures. The solid-state diffusion process forms a fully interconnected porous coating at each point of contact of the fibers [57]. However, the main drawback of this process is that compacting the metal fibers to the form of the implant structure is challenging and time-consuming. Moreover, the interfacial bond between the dense implant core and the fiber mesh coating depends on the complexity of the contours of the implant structure [53]. Even though the sintering method of building porous structures is relatively mature, several limitations exist. A most relevant limitation is that particle oxidation could inhibit the proper bonding of the particles because it is a high-temperature operation. Further, solid-state diffusion bonding of particles usually results in the neck formation comprised of brittle phases and might result in lower mechanical toughness and fatigue resistance. Moreover, the pore size and morphology are usually irregular and largely dependent on the particle size and shape. However, these limitations could be substantially improved upon by using appropriate sintering techniques [13].

Many modified sintering techniques have been investigated to produce porous metallic structures with improved porosity and controlled pore morphology, including the space holder, the spark plasma sintering (SPS), and the replication methods. In the space holder method, several investigations have used carbamide particles as the space holder in preparing porous Ti and porous Ti6Al4V alloys. Carbamide has been chosen in the space holder method because of its ideal spherical particle geometry and chemical properties, such as ease of removal before sintering [58, 59]. A properly sieved and sized mixture of Ti or Ti6Al4V with carbamide was weighed and compacted under pressure. The compaction was then heat treated so that the carbamide particles dissociated at lower temperatures (∼193 °C), and the dissociated by-products were expelled by either using a vacuum furnace or by the continued flow of argon. Thereby, the heat treatment cycle was typically followed where the compact was first heated up to 100 °C, and then slower heating rates were used up to 500 °C to ensure enough time for most of the carbamide particles to dissociate, and the consequent by-products were expelled. Following this, much faster heating rates could be used up to the sintering temperature at which the compaction was held for a considerable time, depending on the size of the compaction being sintered [58]. In this method, the size, shape of the pores, and porosity could be controlled primarily by controlling the volume fraction and the shape of the space holder particles. Other parameters that determine the porosity and the pore morphology are the compaction pressure and the holding time during sintering. While the compaction pressure applied to prepare the pre-sintered compact varied between investigations, the dependence on the mean porosity and mechanical properties (such as yield strength) and the compaction pressure seemed similar, i.e. as the compaction pressure increased, the mean porosity decreased. Also, it was observed among several investigations that the porosities that could be produced by this method ranged between 55% and 75%. It was observed that most of the pores were considered to possess consecutive and open cell morphology, with the sizes of the majority of these pores (<700 *μ*m) being less than the largest space holder particle size (∼700 *μ*m). The pores with larger sizes were observed to be the consequence of pore coalescence. Furthermore, the pore walls’ thickness is believed to significantly impact the porous structure’s mechanical strength. Thereby, it has been observed that, while the pore wall thickness of the structure produced by this method is in the same range as any other powder metallurgy process (i.e. ∼100–200 *μ*m; compared with Berger’s report), interconnected angular-shaped micropores were observed along the pore walls suggesting that the sintering process was incomplete because of low diffusivity. While these micropores were considered beneficial in improving the interconnectivity of the pores, they might significantly deteriorate the structure’s mechanical properties. Different space holders or compaction techniques investigated other variants of this process [60, 61].

**Figure 5.**
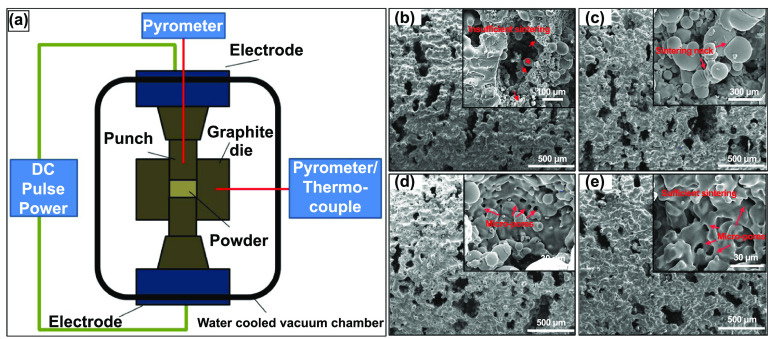
Field-assisted sintering technology/spark plasma sintering (FAST/SPS) technique and the produced porous structures. (a) A FAST/SPS apparatus displaying the working principle. [62] John Wiley & Sons. © 2014 The Authors. Advanced Engineering Materials Published by WILEY-VCH Verlag GmbH & Co. KGaA, Weinheim. Additionally, SEM images of pores walls of porous Ti produced at pressureless conditions with varying SPS sintering temperatures: (b) 1000 °C, (c) 1050 °C, (d) 1100 °C and (e) 1200 °C. Reprinted from [65], Copyright (2015), with permission from Elsevier.

Another variant of the sintering technique is the SPS method. SPS or field assisted sintering technique (FAST) is a process similar to hot pressing where the heat required to sinter the powder particles in a compact is provided by joule heating due to the current flowing within the compact [62]. The general working principle of SPS, as indicated by figure 5(a), is that the sintering powder is compacted within a graphite die and DC voltage pulses pass through the die, and the compact (in the case of conductive sintering powder) produces joule heating, with heating rates up to 1000 °C·min^−1^, cooling rates of up to 400 °C·min^−1^ and maximum temperature of 2400 °C, sintering conditions could be facilitated by appropriately controlling the pulse voltages and durations [63]. Furthermore, this process could be controlled by controlling the die’s measured temperature, power, or current. The rapid heating and cooling rates make this one of the fastest sintering processes, thereby ensuring limited grain growth in the sintered metal [64].

The powder mixture is produced similarly to the space holder technique to produce porous metal structures using the SPS technique. The metal or alloy powder is mixed with varying volume fractions of space holder constituents such as NaCl or NH_4_HCO_3_. Then, the powder mixture is cold compacted under pressure to produce green compacts, sintered using SPS in a specially designed graphite die, as shown in figure 5(b) [65]. In the case of the study, where NaCl was used as an additive, post-sintering dissolution of NaCl in deionized water produced porous Ti6Al4V structures [66]. And in the case of the study where NH_4_HCO_3_ was used as an additive, it dissociates into NH_3_, H_2_O, and CO_2_ and is expelled during the sintering process and thus produces porous Ti structures [65]. In both studies, the x-ray diffraction (XRD) analysis of the porous structure showed negligible impurity content indicating that the space holders used were eliminated during the process. Also, it was observed that the majority phase appears to be *α*-Ti in the post-sintered structure, and the grains developed in the sintered porous structure are mostly fine. This indicates that the SPS process offers extremely fast heating and cooling rates.

The porosity, pore size, and morphology of the porous structures produced by the SPS method showed similarity with the previously discussed space holder method. In general, two types of pores were observed: Macro pores caused due to the dissociation of the space holder, and the micropores, within the pore walls of the macro pores, caused because of the incomplete sintering between adjacent metal particles (as shown in figures 5(c) and (e) in both the studies). Both studies also observed that as the sintering temperature was increased or by heat treatment post-sintering, the pore walls became thicker, and the porosity reduced (as shown in figures 5(d) and (f) in both studies). Also, both studies produced 40%–70% porosities; most pores were well interconnected.

**Figure 6.**
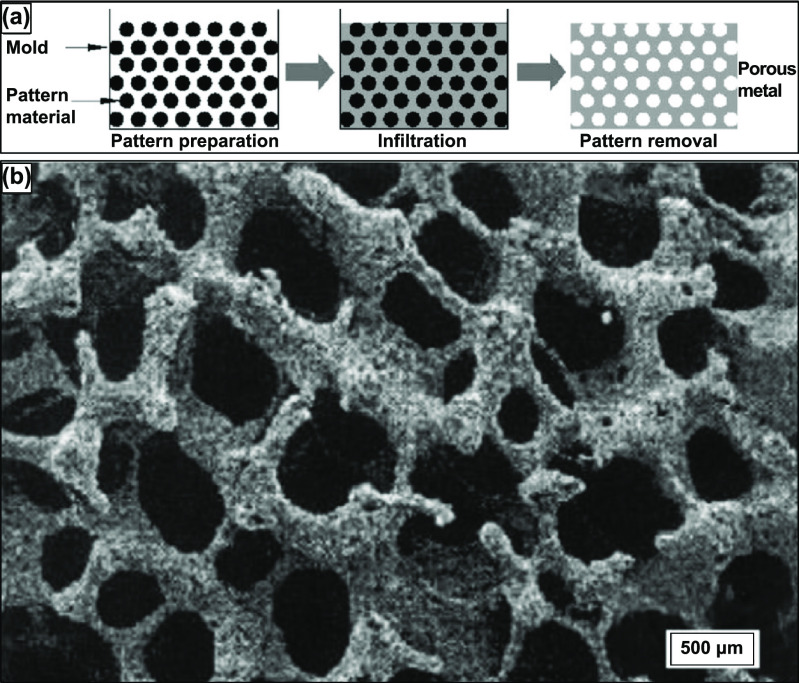
Schematic representation of the (a) 3-step replication sintering process, (b) SEM micrograph of reticulated Ti-6Al-4V foam pore morphology of 88% porous Ti alloys. Reprinted from [13], Copyright (2006), with permission from Elsevier.

Finally, the replication method’s third variant of the sintering method is discussed here. This process is typically three-step and has traditionally been used to prepare porous ceramics [13]. However, some investigations have used this method to produce porous Ti and Ti alloys. This method immersed polyurethane foams in a slurry and then rapidly dried for the metal powder to positively maintain the polyurethane foam replica. The slurry comprises 70% by weight Ti6Al4V and 20% by weight H_2_O and ammonia solution, where the ammonia solution has been added to improve the flow properties of the slurry. After repeating this process multiple times till the polyurethane foam struts were coated entirely with the Ti6Al4V powder, the polyurethane foam and binder were removed thermally, and the remaining Ti6Al4V powder arrangement was sintered, forming an open-cell reticulated Ti6Al4V foam [67]. This three-step process’s schematic is shown in figure 6(a) [13].

As expected, in this process, the flowability of the slurry, controlled via particle size distribution, binder chemistry, the pH of the slurry, air bubble quantity, and the solid–liquid ratio, determines the quality of foam produced by this process [68]. By this process, Ti alloy foams of 88% primarily open porosity have been attained, and the pore morphology could be observed in the SEM image shown in figure 6(b) [67]. The pores formed are chiefly found to be of three types: the primary porosity, comprising of smaller pores on the strut surface; secondary porosity, medium-sized pores formed at the core of the hollow strut formed by previously occupied polyurethane foam; and the tertiary porosity, larger open pores between the struts. A subsequent study observed that a second coating of the powder slurry and second sintering on a previously sintered foam enhanced the density and mechanical properties of the Ti or Ti alloy foams [69].

Even though sintering is a very mature metallurgical process, up to 80% of porosities could be achieved with slight modification. However, the process has inherent limitations. Sintered porous metallic structures are susceptible to brittle fracture and low fatigue resistance. Since, in this process, the metal particles bond via a solid-state diffusion process, the neck formed between metal particles after sintering is usually quite brittle. Also, the sintering process usually produces non-homogeneous pore distribution and pore morphology.

#### 2.2.2. Combustion synthesis.

Combustion synthesis (CS) or self-propagating high-temperature synthesis (SHS) are commonly used with oxide-based materials. Typically, in conventional SHS, condensed-phase combustion takes place using solid-state reactants. A derivation to SHS would be solution-CS (SCS), where the initial reaction medium required is aqueous-based [70]. Additionally, if nanoparticle synthesis occurs in a flame, it is called gas-phase combustion. Most commonly, the techniques are used to produce various oxide materials, a wide range of highly pure and homogenous nanocrystalline powders [71]. CS can be accomplished in two differing modes. The first would require preheating by an external source up to the ignition temperature. Once the reaction is initiated, the reacting layer becomes the igniting heat source for the subsequent layer. The second mode is volume CS, in which the entire reactive body is uniformly brought up to the ignition temperature, and the reaction commences homogenously in the reactive body. The process is considered energy efficient as some thermodynamic systems reach temperatures of 500–4000 K with temperature rates of 10^3^–10^6^K·s^−1^ [72]. This process can form alloys Nitinol (NiTi), NiAl, TiSi, etc [73, 74]. Among those alloys, most literature concerning porous metallic orthopedic implants primarily focused on porous NiTi alloy. Thus, in this review, emphasis has been placed on the fabrication of porous NiTi alloy via CS.

The development of porous NiTi alloys via the SHS method requires an equimolar mixture of Ni and Ti powders with comparable particle size distribution to be prepared. The exothermic reaction between Ni and Ti when producing NiTi alloy is a low exothermic reaction (Ni + Ti → NiTi + 67 kJ·mol^−1^). Therefore, to produce a self-sustaining combustion wave to propagate throughout the initial powder compact, preheating to the ignition temperature is required, as previously mentioned [13]. Once the activation energy barrier is overcome, the combustion wave propagates throughout the green compact and is self-sustained [75]. In this case, a preheating temperature of approximately 450 °C produced a combustion temperature of 1260 °C. It has also been observed that finer powder particles tend to produce higher combustion temperatures for a specific preheating temperature. Important and contrasting observations made on the pore distribution and the combustion temperatures between two similar investigations were that by altering the preheating or ignition technique of the green compact, much higher combustion temperatures were obtained, and the pore distribution was observed to be more isotropic compared to the anisotropic porosity of the conventional preheating method [76].

This method’s resulting crystallographic phases most commonly produced were B2 (NiTi) and B19′ (NiTi). Li *et al* observed that up to 57% porosity with about 86% open porosity could be achieved with this method [77]. These results corroborate with Aihara *et al* reported 60vol% porosity for improved cell response. The correlation between vol% and cell size and interconnectivity will be briefly discussed here but more so in the biological properties section of the review. Pore morphology in the NiTi alloy may be tailored by varying the particle size distribution, binder use, compaction pressure, and preheating temperature of the green compact. Porosity due to unforeseen events may be present due to differences in molar volume and Ni and Ti diffusion flux. Gas bubble formation at high-temperature during the combustion phase is another reason [77].

Aihara *et al* work displayed the practicality and advantages associated with the low-cost CS method. Expanding on the fundamental knowledge and research associated with the CS of Nitinol, Aihara *et al* work attained a successful three-dimensional anisotropic structure with open-cell interconnectivity. The produced structure exhibited comparable effective stiffness of cancellous bone at 1 GPa, achieved by producing 60vol% porosity and pore size of 100–500 *μ*m. A homogenous composition comprising cubic and monoclinic NiTi was achieved without observing pure Ni or Ni-rich phases. The results showed increased corrosion resistance, rapid osseointegration within two weeks, and complete bone growth in six weeks.

#### 2.2.3. Vapor deposition.

**Figure 7.**
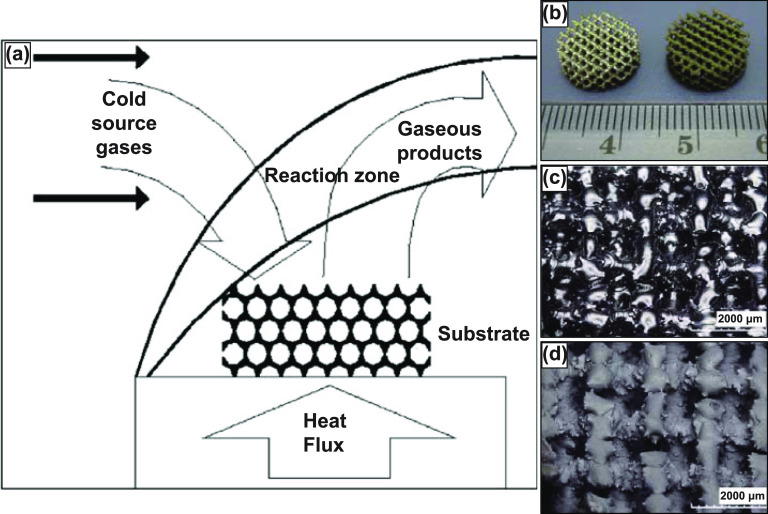
The chemical vapor deposition (CVD) process and produced porous structures. (a) Schematic of the vapor deposition process. Reprinted from [13], Copyright (2006), with permission from Elsevier. (b) Porous Ti6Al4V scaffolds without tantalum coating (left) and with CVD tantalum coating (right). Optical high magnification micrographs depicting the porous Ti6Al4V structure (c) before coating and (d) after CVD tantalum coating. Reprinted from [82], Copyright (2013), with permission from Elsevier.

Vapor deposition is a relatively new metallurgical process for producing high-porosity metal structures with homogeneous porosity. The method involves using medical-grade polyurethane foam to produce a low-density reticulated vitreous carbon skeleton (RVC) by reticulation and pyrolyzing. These RVC skeletal structures are then machined and shaped into preforms, determining the pore morphology of the final porous structure. Then, using a specialized chemical vapor deposition/infiltration (CVD/CVI) technique, tantalum (commercially pure) is deposited throughout the RVC preforms [78]. In CVD/CVI process, generally, reactants in the gaseous phase are activated such that they chemically react, and solid material is deposited on a substrate. The process is schematically illustrated in figure 7(a). The reactant gases and carrier gas enter the reaction chamber at room temperature and are then heated via radiation close to the deposition surface or a heated substrate [78]. The heated reactant gases homogeneously undergo a chemical reaction in their vapor phase before striking the substrate and being deposited. However, this reaction and the products of this reaction are strongly dependent on the process and operating conditions. Ultimately, the reaction products are deposited on the substrate, and the gaseous by-products are eliminated from the chamber [13].

The porosity and the mechanical properties of the structure produced by this method depend primarily on the duration of the CVD process. Typically porosities produced by this method are in the range of 75%–85% (as in Trabecular metal) with an average pore diameter of 550 *μ*m [79]. The scaffold-like Ta structure obtained via this method is comparable to that of the cancellous bone in open interconnected porosity and strut thickness, observable in figures 7(b) and (c). The porous structure obtained by this method displayed a 99% Ta and 1% vitreous carbon by weight presence [79]. Further investigations of the mechanical properties of porous Ta produced via this method indicated that the material’s strength, stiffness, and flexural rigidity increased with increasing density [80]. Ta has been under continual investigation as numerous studies have reported that the mechanical properties of porous Ta far exceed those of other biocompatible metals. Moreover, the porous Ta structure showed superior ductility to other porous materials [81].

#### 2.2.4. Additive manufacturing.

Rapid prototyping or 3D printing is a relatively novel manufacturing process that has proven to produce porous implants of varying sizes, shapes, and porosity—all tailorable directly from the computer-aided design (CAD) to the machine with minimal to no tooling requirement [83, 84]. Additive manufacturing (AM) techniques used to produce porous biomaterials are displayed in table 3, along with details associated with the most common vendor, process, and material details [85, 86]. In the case of porous metallic orthopedic implants and scaffolds, AM-based processes lead the recent advances. Various AM processes are currently being used to manufacture metallic implants, figure 1 [13, 86]. The AM technologies have significantly contributed to porous scaffolds to enhance biocompatibility [87–89].

**Table 3.** Various additive manufacturing methods, commercially available techniques, processing details, and materials.

3D printing techniques	Commercial technologies/vendors	Process details	Processing materials	Advantages/disadvantages [90]
Vat Polymerization	Stereolithography by 3D systems	Layer-by-layer fabrication by curing thermoset photopolymer via exposure.	Mostly photocurable, thermoset polymers	High level of accuracy and finish, relatively quick, relatively large build areas and model weights (200 kg)/relatively expensive, time-consuming post-processing of resin removal, limited photo-resins, requires support structures and post-curing
	Bioplotters by Envisiontec			
	Large Area Maskless Photopolymerization by DDM Systems			
	Lithography-based ceramic manufacturing—Lithoz			
Material Extrusion	Fused Deposition Modeling by Stratasys	Layer-by-layer deposition via extrusion of usually thermoplastic polymers or polymer-ceramic composite.	Various polymers and ceramics include Polycaprolactone, Hydroxy Apatite (HA), Polylactic Acid, Bioglass, Polyethylene Glycol (PEG), etc.	Very common process and inexpensive, suitable for prototyping/nozzle radius limits the part quality, accuracy, and production speed are low for larger parts
Powder bed fusion	Selective laser sintering by 3D Systems	Layer-by-layer selective sintering or melting of powder particles on a powder bed system via laser- or electron-beam-based heat source.	Many metals or alloys such as Ti/T alloys, Stainless Steel, Ni-Ti, etc. Ceramics were also reportedly used.	The powder acts as a support structure for intricate part fabrication, a variety of material options/
	Electron Beam Melting by Arcam			
	Direct Metal Laser Sintering by EOS			
	Selective Laser Melting from SLM solutions			
Directed energy deposition	Laser Engineered Net Shaping by Optmec Inc.	Powder particles are simultaneously flowed through a nozzle and fused using a heat source (Laser, wire arc, and Electron Beam)	Many metals or alloys such as Ti/T alloys, Stainless Steel, Ni-Ti, etc. Ceramics were also reportedly used.	Ability to control grain structure, low raw material loss/may require post-processing for desired surface finish, warping with larger/taller structures
	Direct Metal Deposition by DM3D			
	Electron Beam Welding by Sciaky Inc.			
Material Jetting	Objet by Stratasys	Material is jetted through an ink jet type nozzle, continuously or Drop on Demand (DOD), and solidifies on a substrate, building the part layer-by-layer.	A wide variety of polymers and ceramics can be used.	High accuracy and low material waste, multi-material and varying color capabilities/support material required; only polymers and waxes may be used
	Solidscape 3D printers from Solidscape			
	Multi-Jet Fusion Technology by HP			
Binder Jetting	ZCorp	Like material jetting, a binder is jetted selectively onto a powder bed, fusing the desired powder particles, which lowers to accommodate the next layer of powder.	A wide variety of polymers and ceramics can be used.	Varying color capabilities, metals, polymers, and ceramics may be used, large binder-powder combinations for varying mechanical properties/high porosity in metals, post-processing time can be significant
	ExOne			
	Voxeljet			
Sheet Lamination	MCor Technologies	Thin sheets of material are bound together and ultrasonically welded together. This process continues layer by layer.	Metallic sheets could be used to build simple geometries.	Low cost and fast, ease of material handling/strength and integrity rely on adhesive, limited material usage

The general advantage of using rapid prototyping processes over conventional powder metallurgy processes in fabricating porous metal structures is that powder metallurgy, particularly sintering, produces brittle structures with limited control over pore size and shape, volume fraction, and distribution. Moreover, some conventional processes using foaming agents or molten metal generally result in contaminants and impurity phases [91]. Thus, 3D printing or AM has shown to be the future for producing patient-specific implant structures with controlled shape and porosity. Studies investigating porous metal-based rapid prototyping will be elaborated for some of the more common methods.

##### 2.2.4.1. Powder-bed-based metal AM methods.

**Figure 8.**
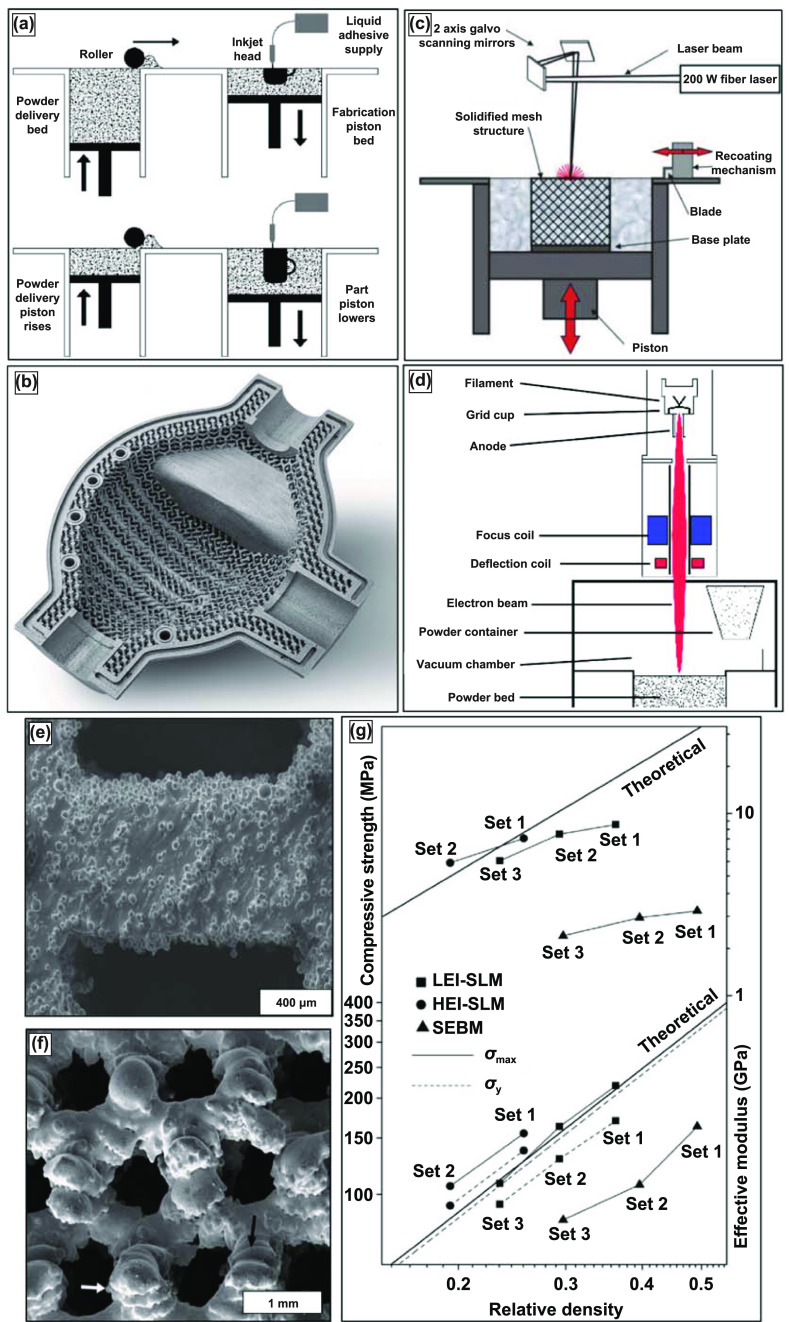
Binder jetting, selective laser melting (SLM), and electron-beam melting (EBM) process with produced structures and compressive testing data. (a) Schematic of the binder jetting process for manufacturing porous metallic structures. Reprinted from [13], Copyright (2006), with permission from Elsevier. (b) Typical surface resolution of a part built using the binder jetting process. Reproduced with permission from [98]. (c) Schematic representation of SLM and EBM setups for [92] John Wiley & Sons. Copyright © 2008 Wiley Periodicals, Inc. and (d) Reprinted from [99], Copyright (2016), with permission from Elsevier respectively. (e) Reprinted from [96], Copyright (2013), with permission from Elsevier and (f) display microstructures of porous Ti6Al4V samples prepared via SLM and EBM, respectively. [97] John Wiley & Sons. Copyright © 2011 Wiley Periodicals, Inc. and, (g) comparative mechanical properties of porous Ti6Al4V samples prepared via SLM and EBM processes. Reprinted from [96], Copyright (2013), with permission from Elsevier.

Several variations of powder-bed AM exist—binder jetting, selective laser melting (SLM), and electron beam melting (EBM). Binder jetting, displayed in figure 8(a), uses print head technology similar to ink-jet printing; the binder selectively prints the part layer-by-layer with a binder. This process is repeated until the entire part is completed, commonly called the *green part*. The green part is then pulled from the print bed, de-powdered, and fired for binder burnout and densification. The binder gives the green part sufficient structural rigidity for de-powdering and firing. Porous surface metal implants with extremely controlled surface porosity and texture, as shown in figure 8(b), can be obtained [13]. SLM and EBM, displayed in figures 8(c) and (d), respectively—unlike binder jetting—rely on thermal energy input during layer-by-layer production for part fabrication. SLM utilizes a laser source to selectively melt and bond the metal powder particles, whereas the EBM method uses an electron beam source—most commonly from a tungsten filament. SLM is usually performed in an inert Argon atmosphere, while EBM is conducted under high vacuum conditions. The benefit of fabricating under a vacuum in the EBM method is a significant reduction in oxidation products and impurities, in general. The three variations of powder bed AM produced porous metal structures of complex geometries with controlled internal architectures.

The schematic of the SLM method has been indicated in figure 8(c), and it should be noted that this method is suitable for any processable powder, either elemental or alloy, with ideal flowability [92]. The parts could be built layer-wise on a substrate that could be moved vertically downwards using a scanning laser source in an argon environment (<0.2% O_2_ levels). The powder exposed to the scanning laser is fully melted, and depending on how high the laser energy density is, either fully dense or porous metallic structures could be manufactured. Furthermore, it should be realized that the best results of this process could be obtained from powders with better laser absorbability and lower thermal conductivity. Higher laser absorbability of the powder ensures lower laser energy density required to melt the powder, and lower thermal conductivity implies that the melt pool remains small and concentrated in the region where the laser is directed [92].

In the case of the EBM method, as shown in the schematic in figure 8(d), the tungsten filament housed in an electron beam head reacts with excited electrons resulting in an electron beam [93]. Then, using two different magnetic fields, the electron beam is first organized into the desired shape and then focussed towards the target position on the metal powder on a powder bed that is retractable, and the part is built layer by layer as per the sliced information of the CAD model. The entire part-building process usually occurs under a high vacuum, ensuring low oxidation and low impurity phases. SLM and EBM processes require post-processing to blow or clean the excess unbonded metal powder particles [94, 95].

SLM and EBM processes can produce structures with up to 80% volume porosities. The mechanical properties can be tailored by varying the process parameters, including energy input and part orientation to the main loading direction. The SEM images of the microstructure of the porous Ti alloy structures built using SLM and EBM are shown in figures 8(e) and (f), respectively. These images show partially melted metal particles sintered at the surface [96, 97]. In one of the studies, which compares the mechanical properties of SLM-made and EBM (or also SEBM) made porous Ti structures, it was observed that there is a considerable difference in the mechanical properties of structures of similar porosity. SLM-produced porous structures were observed to have much thinner struts than the designed dimensions, whereas EBM produced much thicker struts than the intended design thickness. This implies that the SLM-made structures have higher relative porosity for the same designed structure than the EBM-made structure [93]. Also, the microstructural comparison showed that the SLM-based structures comprise *α*′ phase (martensitic), whereas the EBM-based structure comprises *α* + *β* equilibrium phases [96].

A comparison of the effective stiffness and the ultimate compressive strength between SLM and EBM-made and also theoretical stiffness and strength of porous Ti structures of similar relative density (as shown in figure 8(g)) shows that SLM-made Ti structures have improved mechanical properties compared to the EBM-made porous Ti structures and similar (or slightly lower) than the theoretical values [80, 93, 96]. A similar conclusion has been obtained for the comparative investigation conducted for porous Ti6Al4V structures made from SLM and EBM methods. EBM-processed structures tend to show higher ductility despite lower stiffness and strength properties. These mechanical property differences between SLM and EBM-made porous structures could be attributed to the difference in the microstructural phases induced by each process.

##### 2.2.4.2. Directed energy deposition (DED) of porous metallic materials.

The DED technique eliminates the need for a powder bed; the raw material—metal powder or metal-wire filament—is fed into the melt pool created by a focused energy source. The energy source can be a laser, electron beam, or a current-discharge induced electrical arc, which is the case with wire-based DED. Currently, there are many variants of the DED technique that are commercially available, which largely depend on raw material (powder-based or filament-based), energy source (laser or electron beam-based), or CNC control (substrate controlled or source controlled) [100].

**Figure 9.**
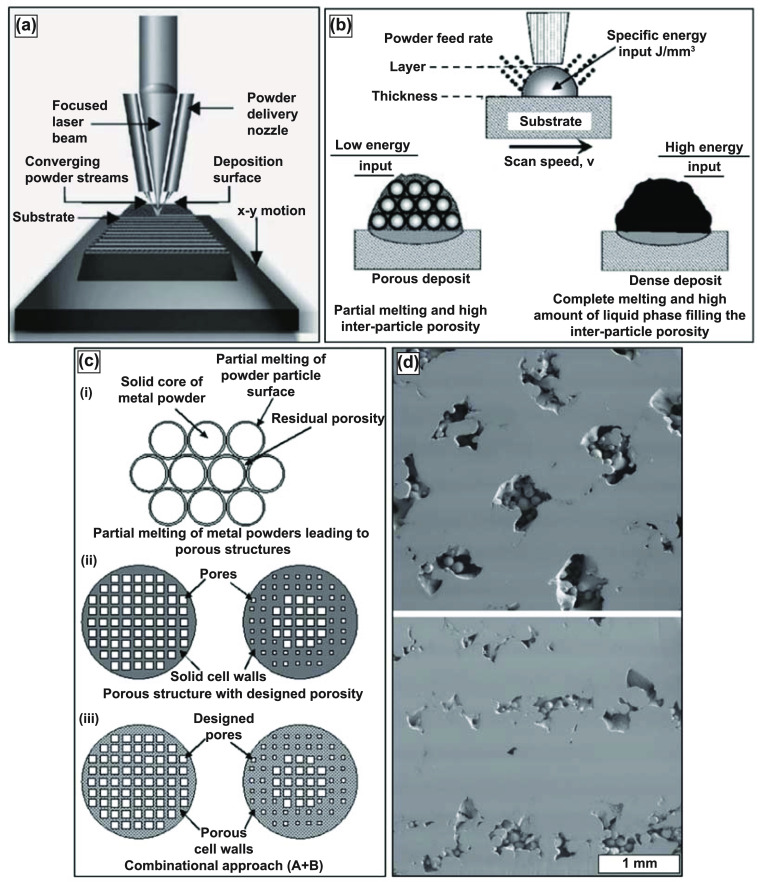
Directed energy deposition (DED) technique displaying the porosity variability. (a) Schematic of DED-based metal additive manufacturing setup; (b) schematic for fabrication of porous structures using DED. Reproduced from [101], with permission from Springer Nature. (c) Design concept to fabricate complex or functionally graded structures using DED by (i) partial melting, (ii) designed porosity, (iii) combination approach, and (d) transverse and longitudinal micrographs of LENS^TM^ made porous Ti compacts. Reprinted from [16], Copyright (2007), with permission from Elsevier.

Sandia National Lab first developed the blown-powder DED technique to manufacture a metal part directly from a CAD file. The part deposition begins with directing a focussed laser beam, usually Nd-YAG, on the metal substrate to produce the melt pool; at the same time, metal powder is injected directly into the melt pool through a coaxial deposition head by the use of carrier gas, as displayed in figure 9(a) [101]. Compared to conventional sintering methods, one advantage of the DED technique is the ability to either entirely melt or sinter fuse the metal particles at a fraction of the time and thermal energy requirement. Bonding between metal particles is formed in the molten state in contrast to the solid-state diffusion-based bonding observed in the sintering process. Furthermore, from the deposition methodology, it has been reported that the cooling rates for this process are extremely high—in the range of 10^3^–10^5^K·s^−1^. The high cooling rates promote solid-state phase transformation and the formation of a supersaturated solution with non-equilibrium phases. Very fine microstructures are formed, having extremely low elemental segregation and fine second-phase particles. Moreover, by altering the process parameters such as the laser power, layer thickness, powder flow rate, scan speed, scan pattern, etc, the effective modulus, porosity, and several other mechanical properties can be altered. Additionally, considering the fabrication process occurs in an inert atmosphere, compositional purity is unaltered [101].

Controlling the porosity can be done in two ways—(1) by introducing inter-particle porosity or (2) by varying scanning strategy. By altering the process parameters, the total energy input—energy density—into the melt pool may be controlled; lower thermal energy density facilitates partial melting of the metal particles, allowing for inter-particle porosity. On the other hand, the toolpath porosity is scanning strategy-based, most commonly allowing for physical separation of independent beads of deposited material, i.e. hatch spacing. The scanning strategy allows for fabricating functionally graded porosity or precise internal porosity architecture. Illustrations for the fabrication of porous parts using a commercial DED process, known as laser-engineered net shaping or LENS^TM^ are displayed in figure 9(b) [101], and a design concept for building complex or functionally-graded porosity is shown in figure 9(c) [16]. Such fabrication feats have allowed for the advancement of metallic biomaterials for patient-specific implantation sites.

A high volume of published literature investigates LENS^TM^-based porous metal structures’ mechanical, biomedical, physical, and chemical properties. For instance, LENS^TM^-made porous Ti or Ti6Al4V structures were studied to understand the dependence of the stiffness and the strength of the material on the porosity and the pore morphology. As discussed above, the material’s porosity depends chiefly on the process parameters and scanning strategy. It was observed that about 20%–70% highly interconnected porosity, with >90% open porosity, could be achieved using lower laser power, higher scan speed, higher hatch distance, and higher powder feed rate—equating to low powder mass flow rate and low thermal energy density. Micrographs of LENS^TM^ transverse and longitudinal sections made of porous Ti parts are displayed in figure 9(d). The micrographs displayed preferential pore connectivity in the longitudinal build direction than in the transverse orientation for parts with lower porosity. However, as the porosity increases, pore interconnectivity becomes more uniform [16]. Another critical relation between the various process parameters of the LENS^TM^ method and the porosity is that while the laser power, scan speed, or the powder feed rate affect the porosity, the pore size remains unaffected by these parameters and is singularly influenced by the hatch distance; that is, as the hatch distance increases, an increase in mean pore diameter occurred [42].

#### 2.2.5. Fiber mesh-based porous implants.

An additional AM method for fabricating porous coatings on implant surfaces for enhanced cementless implant fixation is via sintering fiber meshes, comparable to the sintering of powder metals. The advantage of using fiber meshes instead of metal powders is that the porosity of the coatings obtained through this method is almost always interconnected, enhancing bone ingrowth and implant fixation [13]. In this method, the metal fibers are usually compacted onto the implant surface, requiring a biocompatible coating, while the sintering process follows. During the sintering process, metallurgical bonds are formed at points of physical contact between the fibers and the implant surface—attaining sufficient mechanical strength. However, the major disadvantage of using fiber mesh is that it is unfeasible to prepare fiber mesh compact on the implant surface using sufficient compaction forces for complex implant shapes. Furthermore, fiber mesh loss of contact with the substrate due to relaxation post-compaction is quite common, leading to regions of poor bonding with the implant surface. Regarding mechanical stability, the literature suggests that fiber mesh coatings show improved stability than powder-sintered coatings; however, if the failure mode is observed, fiber mesh coatings fail via tearing instead of crack propagation [53]. Also, the porosities of these fiber mesh coatings are usually limited between 30%–50%, which influences the maximum possible strength of the implant fixation via bone ingrowth and is usually attained by non-homogeneous porosity. Although the literature on sintered fiber mesh coatings is available for both stainless steel and Ti, only Ti fiber mesh coatings are available clinically [53–57, 102].

**Figure 10.**
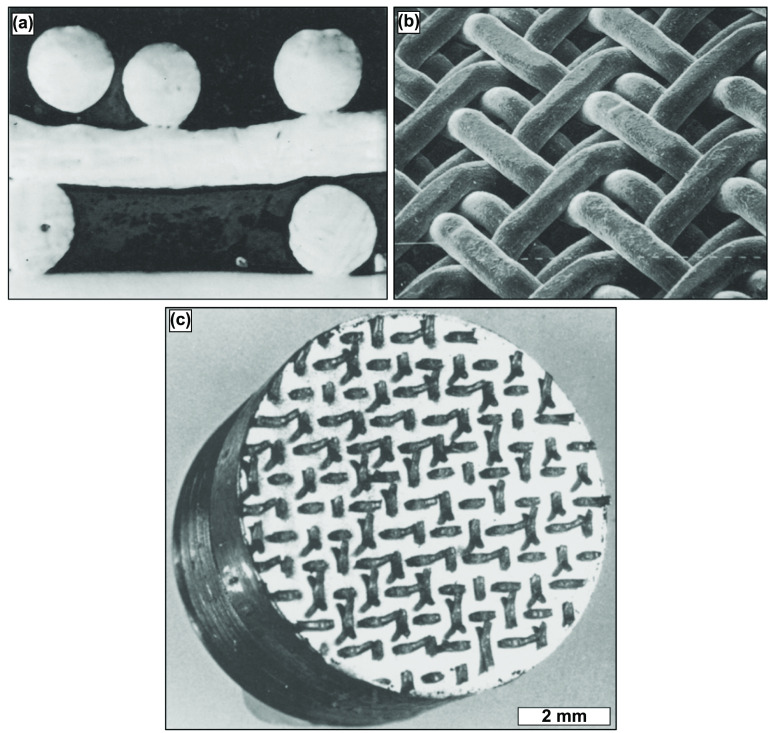
Twill-weaved triple orderly-oriented wire mesh (OOWM) process for porous coating production. (a) Cross-sectional micrograph and (b) SEM image of twill-weaved triple OOWM coating, in which the mesh size is 16 and wire diameter is 500 μm. Reprinted from [103], Copyright (1986), with permission from Elsevier. Displayed in (c) is a Ti OOWM-coated sintered sample post-tension testing revealing that failure did not occur at the interface. Reprinted from [104], Copyright (1986), with permission from Elsevier.

A variant of fiber mesh sintering for producing homogenously distributed porous coatings is orderly-oriented wire mesh (OOWM). This method uses woven continuous long metal small-diameter fibers instead of short fibers for compaction to form an orderly meshwork. The woven fiber mesh is pre-compacted to improve contact with the implant surface and then pressure sintered, analogous to hot isostatic pressing (HIP). The advantage of the OOWM method over the fiber mesh sintering method is that the issues, such as fiber detachment from the implant surface, are avoided. Moreover, twill weaves have been used instead of the regular weaved mesh. These are formed by consecutively passing the metal wires above and below two crossing wires, and any two neighboring wires running parallel pass a crossing wire from above to below, as displayed in figures 10(a) and (b). The advantage of using a twill weave lies in the ease of mesh fabrication, and the following coating procedures also become more accessible because of the improved flexibility and formability of the twill-woven fiber meshwork [103]. Additionally, their performance under tensional testing displayed no failure along the interface, as displayed in figure 10(c).

A final method known as ferromagnetic fiber arrays use ferromagnetic metal fibers to fabricate porous coatings; these use a magneto-mechanical mechanism to induce slight deflection into the porous coatings via an applied external magnetic field. These magnetic field-induced strains and deformations are believed to stimulate bone ingrowth into the pores—as reported, a minimum of 0.1% strain is necessary to stimulate bone growth into the porous coating. Thus, metal implants coated with highly porous, bonded ferromagnetic fibers under an external magnetic field enhance implant-host tissue fixation. In this method, the porous coatings were produced by spraying fibers with slow-setting aerosol glue, surface dispersed brazing powder, and then placed in a long quartz tube at 1200 °C [105]. The coating thus produced high porosities ranging from 70%–90%, with pore size ranging from 100–300 *μ*m. Moreover, ferromagnetic materials also tend to have better corrosion resistance in a biological environment than non-ferromagnetic materials [106–108]. Such properties are highly sought in porous metal implants as corrosion by-products can adversely affect the physiological environment.

## 3. Properties of porous metallic materials

When considering all the fabrication methods for the production of porous metallic structures, porous coatings, or functionally graded porous structures (discussed in the previous section), it can be understood that different pore shapes, sizes, and interconnectivity could be obtained. However, the type and volume fraction of porosity governs several inherent characteristics of the porous structure and its applicability as an implant material. It was discussed in the earlier parts of this review that one of the significant advantages of porosity in metallic implant structures is that it allows bodily fluids to flow through and hence facilitates bone ingrowth which would provide proper anchorage to the implant with the host tissue, thereby increasing the lifetime of the implant. However, this bone tissue ingrowth mainly depends on the pore size and morphology.

It was generally observed that the pore shape had limited biological response [101], whereas pore size and interconnectivity primarily affect the bone tissue ingrowth capability [50, 109–116]. It was accepted that interconnected pore sizes in the 100–400 *μ*m were required for optimal bone ingrowth [50]; however, some bone ingrowth was also observed with pore sizes as small as 50 *μ*m [115, 116]. In the case of substantial pore sizes (>1 mm), there seemed to be a higher possibility of forming fibrous tissue, which is detrimental to the implant’s lifetime as it would eventually cause aseptic loosening of the implant. Another advantage of using porous metallic structures as orthopedic implants are the reduced structure stiffness. As discussed earlier, a much stiffer implant material induces stress shielding, weakening the surrounding host tissue and causing implant loosening. Thereby, a porous metallic implant would have a lower stiffness than a dense implant, and its stiffness could be modeled by altering the porosity and pore morphology such that the implant stiffness is in the same range as the surrounding bone stiffness.

Porosity in a metallic implant structure also introduces inherent limitations in the mechanical behavior of the load-bearing implant structures. The implant’s mechanical strength, dynamic load-bearing capabilities, and corrosive properties are expected to be negatively affected due to porosity. Also, the porosity predominantly affects the implant structure’s biological compatibility. Thus, numerous investigations have been conducted to understand and quantify the effect of porosity on the mechanical behavior of porous metallic orthopedic implants, and in this section, the results, findings, and inferences of various studies investigating the physical, mechanical and biological properties of the porous metallic structures would be discussed.

### 3.1. Physical properties

Porous metallic structures’ physical properties include porosity, density, and hardness. The methods of measuring these properties and the reported values for these physical characteristics will be discussed in detail in this section.

#### 3.1.1. Porosity and density.

The porosity and density of a structure can be used interchangeably, in most cases, depending on engineering vernacular. This is because as a structure’s porosity increases, the structure’s density decreases and vice versa. Moreover, as discussed previously, porosity has two types: open- and closed-cell porosity. If the pores are interconnected and allow for the free flow of fluids, they are called open pores; if the pores are isolated and negate fluid flow, they are called closed pores. One commonly used method for measuring the total porosity is gravimetry. First, the apparent density of the porous structure is calculated by measuring its total volume and weight [65]. Then, the total porosity (*P_T_*) is calculated using the following equation:

\begin{equation*}{P_T} = \left( {1 - \frac{\rho }{{{\rho _s}}}} \right) \times 100\,\% \end{equation*}

where, $\rho $ is the apparent density of the porous structure. ${\rho _s}$ is the bulk density.

The total porosity of the structure combines open- and closed-cell porosity. For orthopedic implants, open and interconnected porosity is desired to facilitate bone tissue growth and improve implant anchorage; it is necessary to know what fraction of total porosity is open porosity. One method used to measure the fraction of open porosity is the infiltration of paraffin into the open pores of the structure by boiling the sample in paraffin in a vacuum chamber. Subsequently, the weight and volume of paraffin penetrating the porous structure are measured. The fabrication method and the processing parameters usually determine the total porosity that can be achieved and the open porosity fraction. However, depending on the metal or alloy being investigated, an optimal porosity could be achieved, making the mechanical properties, such as effective stiffness and strength, comparable to that of the target bone structure. Previously reported, it was observed that a porous Ti structure with a three-dimensionally interconnected open-cell network achieved porosity in the range of 35%–42% and a porous Ti6Al4V structure with porosity in the range of 23%–32%. Both result in mechanical properties similar to human cortical bone [93, 117].

### 3.2. Mechanical properties

Similar to the physical properties desired by orthopedic implants, a load-bearing implant must meet specific mechanical property criteria appropriate for the implantation site. Therefore, it is critical to understand the biomechanics of the host bone site for a proper match with the porous metallic implant for hard tissue replacement or implantation. The skeletal system sustains mechanical loading of several modes with continuously changing loading vectors; such force examples can be simple reaction forces at the hip joint to bending moments and torsional strain along bone segmental bodies. Several investigative studies were conducted on the bone structures to understand the different loading mechanisms endured and the capacity of the load that the skeletal system can endure. Generally, it was observed that the skeletal system experiences loading in uniaxial compressive, flexural, multiaxial, and dynamic loading, such as fatigue or high strain rate [118–121]. Thus, general knowledge of the mechanical properties desired by the porous metallic orthopedic implant has been surmised in literature, which will be discussed.

#### 3.2.1. Hardness.

The hardness of a material is defined as its resistance to localized penetration, scratching, bending, and/or plastic deformation. A material’s hardness is usually measured using an indentation tester under a load. Varying names for the differing methods of hardness exist. Among the most common examples are Mohs, Shore, Brinell, Rockwell, Vickers, and Knoop. The application of each type of hardness testing varies for use in minerals, polymers, and metals and even for thin materials. The hardness number is based on the applied force divided by the surface area of the indent and sometimes assesses the microstructural phase changes of the material [122]. The resulting units are in force per area, but it is not to be mistaken for pressure.

AM-based literature typically uses the microhardness characteristic to quantify the degree of hard phase presence resulting from the fabrication process. DED-processed porous CpTi will typically not display an increase in hardness when processed in this fashion; however, the hardness will increase for LENS^TM^ Ti6Al4V. The presence of aluminum (Al) and vanadium (V) in the Ti matrix influence the stability of crystallographic phase presence at room temperature. Al is an *α*-phase stabilizer in Ti, while V is a *β*-phase stabilizer [123]. The *β-*phase is only present in pure Ti when heated to a temperature above 880 °C; upon cooling, the *β*-phase undergoes a diffusion-less solid-phase change to *α*-phase. Suppose phase stabilizing constituents are present in the diffusion-less transformation from *β*-phase to *α*-phase. In that case, the resulting crystal structure may result in an interstitial atomic-induced strained configuration and thus result in an increase in hardness at room temperature compared to conventionally prepared samples [124]. Therefore, the increase in microhardness of LENS^TM^ processed samples is directly correlated to the fine needle-like *α*′-phase and *β*-phase [125]. Without in-depth materials characterization, hardness testing will provide insight or validation into the crystallographic phase presence.

Although the correlation of hardness increase of Ti6Al4V with LENS TM treatment was discussed, it does not say that varying laser parameters will affect hardness. The contributing factor appears to be simply treatment vs. no treatment. CpTi and Ti6Al4V do not exhibit a change in hardness with the change in laser parameters, even when porosity changes. This indicates that the laser parameters do not influence phase presence, thus allowing biocompatible phases to remain present in structures with varying porosities [124, 126]. Additionally, the hardness of a metallic material decreases as the microstructure becomes coarser, following the Hall–Petch effect; this is because finer grain microstructures tend to create lower stress concentration sites due to dislocation pile up at the grain boundary and thus would require higher stress to deform plastically [127]. Furthermore, it was observed in several studies that the composition of the alloy and the processing temperature influence the hardness of the structure. In the case of processing parameters that influence the phase stability of a material, the hardness value also depends on these parameters. However, in the case of laser-prepared Ti alloys, the process parameters tend to have a negligible effect on the varying phase composition; thereby, the material’s hardness remained unchanged [128]. Microhardness, values of different materials processed via different methods, are reported in table 4 for comparison.

**Table 4.** Microhardness values of various materials investigated by several studies.

Material	Fabrication process	Porosity	Hardness	References
Ta	LENS	—	395 ± 30	[19]
	EDM	—	240–393	[81]
CpTi	Space holder method	—	258–263	[60]
	SPS	—	163	[118]
Ti-6Al-4V	SLM	—	483	[119]
	EBM	—	410–442	[120]
	EBM-High Energy	—	364	[121]
	EBM-Low energy	—	372	
	LENS	10%	251	[23]
		20%	259	
Ti-Mn alloys	SPS	—		[118]
Ti5Mn		—	372	
Ti2Mn		—	245	
Ti12Mn		—	538	
Ti-Nb-Zr alloys	NH_4_HCO_3_ spacer	—		[128]
	2-step foaming process	—		
Ti20Nb15Zr	0% NH_4_HCO_3_	5%	290	
	20% NH_4_HCO_3_	36%	260	
	35% NH_4_HCO_3_	48%	155	
	50% NH_4_HCO_3_	63%	110	
Ti	50% NH_4_HCO_3_	63%	254	
Ti20Nb15Zr		63%	110	
Ti35Nb15Zr		63%	56	
			
Ti20Nb15Zr	1473		290	
	1673		256	

#### 3.2.2. Static mechanical properties.

Static mechanical properties of any material generally include stiffness, creep, yield criterion, uniaxial strength properties, and multiaxial strength properties. Since load-bearing implants are likely to be subjected to compressive loads, ample literature has reported the compressive behavior of different porous metals. Considering that the primary reason for using porous metals in orthopedic implants is to alleviate stress shielding, the effects of porosity and stiffness of the porous structure on the compression strength have been investigated extensively. Furthermore, research has also examined the tensile strength properties and the multiaxial behavior of porous metals. Most of the static mechanical properties reported in the literature and discussed in this section have been enlisted in table 5.

**Table 5.** Critical mechanical performance values for porous metallic biomaterials.

Material	Fabrication process	Porosity (%)	Young’s modulus (GPa)	Compressive yield strength (MPa)	Compressive ultimate strength (MPa)	Flexural yield strength (MPa)	Torsional properties		
							Modulus of rigidity (GPa)	Yield strength (MPa)	Maximum shear stress (MPa)
CpTi	Sintered [51]	5	86.25	250					
		6				725			
		8	76.25	200					
		8.75				500			
		17.5	55						
		20	43.5	160		340			
		28.75	35	100		235			
		32.5	28	60		125			
		33.75	17						
		34				75			
		35		40					
TI6Al4V	EBM [120]	50.75	2.92		163.02				
		60.41	2.68		117				
		70.32	2.13		83.13				
	SLM-Low Energy [121]	60.9	8.73	170	219				
		68.6	7.72	129	163				
		75.8	5.5	93	108				
	SLM-High Energy [121]	74.4	7.3	137	155				
		80.6	5.36	92	106				
	Space Holder Method [60]	62	3.75	52.5					
		63.5	1.9	36					
		66	1.15	22.5					
		71	0.6	14					
		72	0.5	16					
		75	0.26	7					
		75.5	0.25	6.5					
	LENS [23]	0					11	332	455
		3	105	1025					
		10					10	190	235
		17	60	780					
		19	47	760					
		20					5.7	185	233
		23	16	620					
		25	14	580					
		29.5	9	470					
	SPS-Heat Treated [58]	47.6	33	110					
		54.4	22.5	85					
		60.7	16	66					
		70	9	42.5					
NiTi	Combustion Synthesis [77]	8	18		1190				
		12	16		1050				
		18	12		845				
		22	8		580				
		27.5	5		350				
		31	3		220				
		36	2		180				
Ti15Mo5Zr3Al [129–132]	Sintered and Hot-Pressed (HP)	2.5	80			1300			
		10	60			750			
		20	44			310			
		25	22			180			
		33	10			70			
	Sintered and Quenched (STQ)	2.5	79			1350			
		10	50			770			
		25	20			210			
Ti-Nb-Zr	Two-step foaming powder metallurgy method [128]	6	10.8	1550					
		38	8.25	400					
		50	4.2	290					
		62.5	2.5	50					
Ti10Nb10Zr [133]	Space holder sintering method	0	68 ± 3.5						
		42	21 ± 0.3						
		50	7.9 ± 0.7						
		59	5.6 ± 0.3						
		69	3.9 ± 0.3						
		74	1.6 ± 0.2						
Magnesium	Space holder method using carbamide particles [89]	0	43	100		80			
		36	18	34		28			
		44	6	25		21			
		55	4	15		14			

##### 3.2.2.1. Uniaxial mechanical properties.

Porous orthopedic metallic implants must possess an effective modulus comparable to the host bone tissue to avoid premature aseptic implant loosening by stress shielding. Since porosity in metals tends to compromise the structure’s mechanical properties, a fine balance is at play when considering that stiffness and strength decrease with increased porosity—establishing an inverse relationship. For this reason, most literature focuses on determining the ideal porosity for adequate, effective stiffness, mechanical yield, and ultimate strength [134, 135]. An abundance of literature on porosity’s influence on mechanical properties is available, such as for commercially pure Ti or Ti6Al4V. Additional studies focused on metals and alloys such as Mg, NiTi, TiNbZr, Ti15Mo5Zr3Al, etc [51, 59, 66, 93, 96, 128–132]. Thus, this section discusses various materials’ uniaxial-mechanical properties, such as stiffness, compressive strength, tensile strength, percent elongation, etc, to porosity. Alternatively, the dependence of mechanical properties on the fabrication processes is also discussed for some of the previously mentioned fabrication processes.

**Figure 11.**
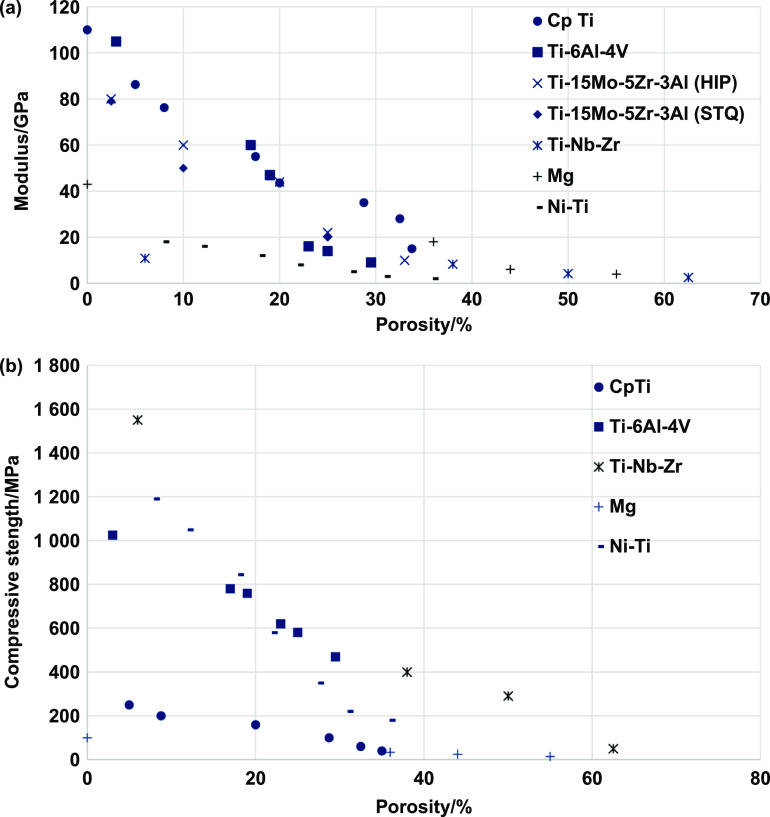
Cumulative plots for (a) Young’s modulus to percent porosity for several metals and alloys, (b) compression strength to percent porosity for several metals/alloys. Values attained and graphed from the following sources [51, 59, 66, 93, 96, 128–132].

The graphs in figures 11(a) and (b) display the effective modulus of elasticity and compressive strength of porous materials, such as commercially pure Ti (CpTi), Ti6Al4V alloy, Ti15Mo5Zr3Al alloy, TiNbZr alloy, Magnesium, and NiTi alloy to porosity. As previously mentioned, an inverse relationship is apparent with the increased porosity percent when measuring effective modulus. Additionally, the appropriate porosity for comparable effective stiffness to match the human cortical bone varies depending on the material.

Cortical bone exhibits density of, Young’s modulus, and compressive strength range of 1.8–2.1 g·cc^−1^, 3–20 GPa, and 130–180 MPa, respectively [118, 119]. Thus, for CpTi samples, the ideal porosity seems to be 30%–40%, where the reported effective Young’s modulus and compressive yield strength range between 10–20 GPa and 40–100 MPa, respectively [51]. In the case of porous Ti6Al4V samples, porosity in the range of 20%–30% was found to show Young’s modulus in the range of 8–30 GPa, and compressive yield strength in the range of 470 mechanical properties of porous titanium compacts prepared by powder sintering 650 MPa, i.e. for a stiffness closer to human bone Ti6Al4V samples showed much higher compressive strengths [59, 93, 96, 129]. When considering the alloy Ti15Mo5Zr3Al, 25%–35% porosity displayed Young’s modulus in the 20–10 MPa. It could also be observed from figure 11(a) that Ti15Mo5Zr3Al alloy samples, which were HIP, showed considerably higher Young’s modulus compared to the solution treated and quenched samples (STQ) for the same porosity. This is because of the formation of *α*-Ti in the *β*-Ti matrix in HIP samples compared to the STQ samples. It was also reported that the solution-treated Ti15Mo5Zr3 Al has comparable strength to the HIP samples because of the introduction of oxygen impurities during STQ [131]. For another Ti alloy Ti20Nb15Zr, it was observed that Young’s modulus was consistently low in the range of 11–2 GPa for porosities ranging from 6%–62.5%, while at the same time, the compressive yield strength remained high in the range of 1550 MPa for 6% and about 50 MPa for 62.5% porosity [128]. To obtain low modulus metallic implants, two methods could be commonly implemented: either to introduce porosity in the implant structure or to introduce alloying elements such as Ta, Nb, and Mo, which tend to produce Ti alloys of mostly *β*-phase which tend to have low elastic modulus [128, 131]. While porous surfaces of implant materials tend to have improved adhesion with the host tissue and thus greater fixation, adding the above-mentioned alloying elements has been shown to reduce the elastic modulus of the alloy while maintaining desirable strength properties; This might lead to preferred mechanical performance and fatigue characteristics. Additional porous metals with modulus and compressive strengths in the range of human cortical bone were found to be magnesium and NiTi alloy with magnesium of porosity ranging from 36% to 55%, showing stiffness in the range of 18–4 GPa and compressive strength in the range of 34–15 MPa and NiTi alloy of porosity 8%–36% showing modulus ranging from 18–2 GPa and ultimate compressive strength ranging from 1190–180 MPa [130, 132].

NiTi alloy, a commonly known shape memory alloy, has several other mechanical advantages, such as recoverable strain and superelasticity. A recoverable deformation of 8% is possible with NiTi alloy, similar to that of natural bone, which could recover about 2% of its deformation. This similarity of NiTi and bone properties allows for its biomechanical performance and, thus, better compatibility. To assess this shape memory effect (SME) of the NiTi alloy, the samples of different porosities were loaded at a strain rate of 0.002 s^−1^ up to a total strain of *ϵ*_t_ (2%, 4%, and 6%), and then the samples were unloaded at the same rate, and the residual strain *ϵ_τ_* was recorded. Then the SME was activated by subjecting the samples to thermal cycling, where the temperatures depend on the samples’ density, and then recording the permanent plastic strain *ϵ*_p_. The recovered strain is then calculated as the difference between the total applied strain and the remaining permanent plastic strain given by *ϵ*_m_ = (*ϵ*_t_− *ϵ*_p_). The dependence of the recoverable strain on the porosity is shown in table 6, and it was observed that 100% of the strain recovery happened for a 2% total strain across all designed porosities. The percentage strain recovery decreased at higher total applied strain, but at least 70% of strain was recovered from all samples. The reduction in the recoverable strain for more porous samples was observed due to possible deformation localization at the pore walls and necks, which could significantly increase the strain experienced in these areas compared to the macroscopically applied strain. The strain recovery for these highly deformed regions reduces the overall recovered strain for more porous samples. The trend was 6% applied to strain a sample with 28% porosity showed a recovered strain of about 73% compared to a 90% recovered strain for samples with 12% porosity. Since bone shows approximately 2% recoverable strain, NiTi alloy with porosity could be considered an ideal bone-replacement material [76, 77, 130, 136–140].

**Table 6.** Recovered strain for several porosities of NiTi alloy for 2%, 4%, and 6% applied strain.

% Porosity	Recovered strain		
	2% applied strain	4% applied strain	6% applied strain
10	96	96	90
13	100	90	—
20	100	92	83
25	100	89	78
28	—	—	75
29	95	86	—

Mechanical properties of porous metallic structures were also observed to depend on their fabrication method. This correlation between the mechanical properties and the fabrication method mainly arise from the microstructural or phase differences introduced into the structure due to various processing methodologies. This variation in mechanical properties was found to be more prominent in Ti alloys because of a large variation in the mechanical properties of *α* and *β*-phases of Ti. The variations in the compressive strength and Young’s modulus of porous Ti6Al4V manufactured via Space holder sintering, SPS, EBM, selective laser sintering (SLS), and LENS methods have been summarized [59, 66, 93, 96, 101, 129].

The effects of varying processing parameters on the mechanical properties of the porous structure could be observed from the properties of Ti6Al4V samples manufactured using SLM both by high energy input (HEI) or low energy input (LEI). It could be observed that for much lower porosities, in the range of 20%–30% LENS^TM^ processed Ti6Al4V samples showed lower modulus similar to that of the human bone (i.e. 10–20 GPa) as compared to most of the other processes, which needed about 50%–80% porosities to attain similar modulus values. Moreover, having lower porosities in LENS-processed samples implied much higher compressive strengths than most Ti6Al4V samples with a similar modulus of elasticity. The most plausible reason for the higher ductility shown by the LENS processed samples is that compared to most conventional fabrication techniques, which incorporate solid-state diffusional bonding, the laser power locally melts the metal particles and bonds them, thus eliminating the inherent brittleness. Another reason for low modulus at lower porosities and higher compressive strengths could be attributed to the rapid cooling rates prevalent in LENS, which ensure the presence of high-temperature stable *β*-phase and finer *α* phase of Ti in the microstructure. It could be observed that HEI samples produced thinner struts and introduced non-designed porosity and thus have higher porosity and lower mechanical properties compared to the LEI parameters in the case of SLM.

The effect of microstructural phases on the mechanical properties of Ti alloys could be observed, where the SLM fabricated Ti6Al4V samples of porosity 70.7% are heat treated via water quenching and furnace cooling. It was reported that the furnace-cooled samples (i.e. slowly cooled samples) showed a typical lamellar microstructure of equilibrium *α* + *β* phases, whereas the rapidly quenched sample has the metastable martensitic α′ phase. Heat treatment had an insignificant effect on the modulus of elasticity, with quenched samples showing a higher modulus of elasticity than the furnace-cooled samples, which indicates the presence of the lower modulus *β* phase. Also, the yield and the ultimate compressive strength of the as-processed and the quenched samples are similar and much higher than the furnace-cooled samples indicating the martensitic phase present in the SLM fabricated samples [96].

##### 3.2.2.2. Torsional and flexural properties.

Compressive strength and stiffness are considered to be the most important mechanical properties of load-bearing orthopedic implants, most of the recent literature on porous metallic implant materials is focused on characterizing these properties. However, the mechanical and physiological relevance of porous material’s behavior towards complex loading mechanisms lacks investigation. Thus, this section summarizes the limited understanding of the mechanical behavior of porous metallic structures under flexural and torsional loading.

**Figure 12.**
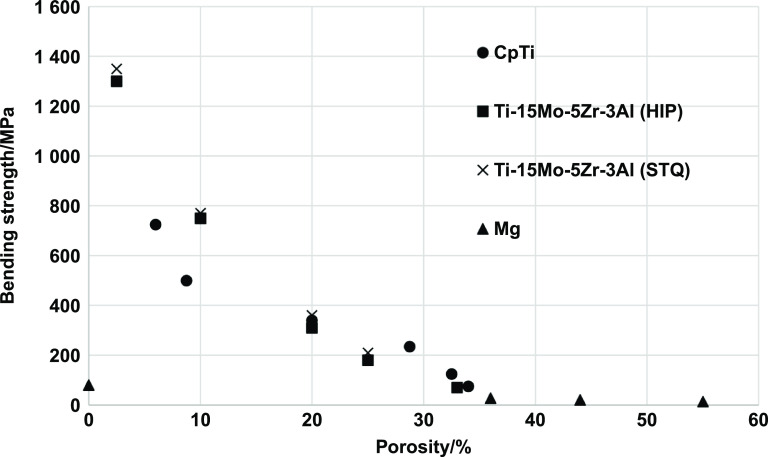
Comparative plots for the bending strength of various materials to porosity values were attained and graphed from the following sources [51, 131, 132, 138].

The flexural behavior of porous metallic implant materials is usually investigated via a 3-point or 4-point bending test using a universal testing machine where samples of standard dimensions are loaded. Correspondingly measured strain is used to calculate the bending stiffness and strengths. In figure 12, the flexural strengths of some of the porous orthopedic implant materials such as CpTi, Ti15Mo5Zr3Al (HIP and STQ), NiTi, and Mg for various porosities were summarized [51, 131, 132, 138]. It could be observed that Ti15Mo5Zr3Al alloy, HIP, and STQ have higher flexural strength properties than any other comparable material porosities. Also, CpTi, with porosity in the range of 10%–30%, has high flexural strength in the range of 100–400 MPa, which is considerably high compared to the flexural strength of the human cortical bone [118]. Therefore, Ti or its alloys would be better for any implant site requiring flexural properties over NiTi alloy or Mg.

**Figure 13.**
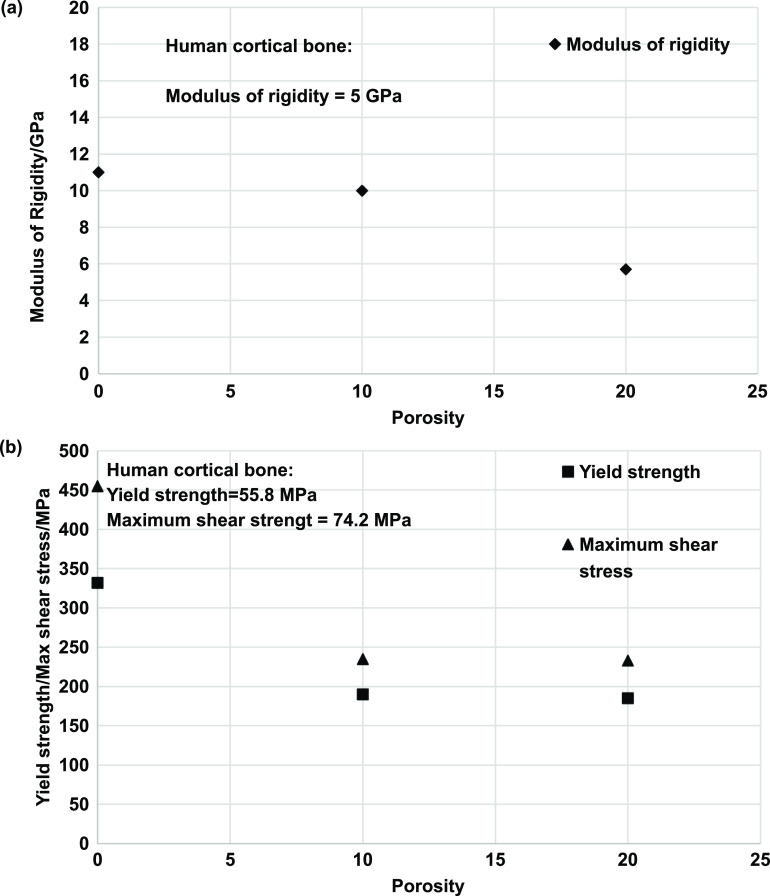
Quasi-static torsional (a) modulus of rigidity vs. porosity and (b) yield strength, and maximum shear stress to porosity plots for porous Ti6Al4V alloy samples. Reprinted from [143], Copyright (2011), with permission from Elsevier.

In order to thoroughly understand the mechanical behavior of porous metallic implant structures under different modes of mechanical loading, it is crucial to consider the torsional behavior of these materials. However, very few studies have investigated the mechanical behavior of orthopedic implant structures under torsional loading. Most of these studies were focused on bulk orthopedic structures [141, 142]. One relevant study that has investigated the torsional behavior of porous metal implants has considered volume porosity influence on torsional deformation of AM processed Ti6Al4V samples with 0%, 10%, and 20% porosity and testing them until failure or 40% drop in torque at the torsional speed of 45°·min^−1^ [143]. Figure 13 shows comparative plots for the torsional properties, such as modulus of rigidity, yield strength, and maximum shear strength.

A 10% porosity in laser-processed Ti6Al4V resulted in a 43%, 48%, and 30% drop in torsional yield strength, maximum shear stress, and shear strain, respectively. At 20% porosity, shear strain at maximum shear stress dropped 58%. Such results show that shear band formation in dense samples requires a higher shear strain. Comparing the yield strength of porous Ti alloy under compressive and torsional loading showed a significant drop during torsional loading. Under uniaxial compressive loading, the porous Ti alloy samples densify due to pore closure, and the stress increases rapidly. However, densification does not occur during torsional loading, and all the stress increases happen because of strain hardening. Because of those differences, porous Ti6Al4V showed faster degradation under torsional loading than in compression. However, stain hardening in porous Ti6Al4V reflects ductile deformation due to strong bonding between the particles due to localized melting and subsequent solidification than sintering common in processing via powder metallurgical route [129]. Naturally, the inherent brittleness can be eliminated in laser-processed samples. Finally, it was observed that the porous Ti6Al4V samples with 20% porosity showed similar rigidity to the human cortical bone, and the yield strength and maximum shear stress for all porosities investigated showed higher strength and stress values than the human cortical bone. Thus, a 20% porous Ti6Al4V sample would have ideal mechanical properties for an implant structure suited for a site with a complex loading pattern.

#### 3.2.3. Dynamic mechanical properties.

By reason of their lightweight and mechanical properties, porous metallic structures are widely used in numerous critical applications such as aerospace, automobiles, biomedical implants, etc. For most of these applications, the dynamic behavior of these porous materials, such as strain rate dependant mechanical strength, energy absorption capabilities, and compression/flexural/torsional fatigue, are considered prominent. Understanding the high strain rate behavior, fatigue, and dynamic mechanical characteristics of porous metals is crucial to design porous metallic structures specific to the applications. In this section, the high strain rate mechanical properties and the fatigue behavior of various porous metals will be discussed in detail.

##### 3.2.3.1. High strain-rate compression properties.

The appeal of porous metallic structures in areas with high-velocity deformation or a high strain loading rate is mainly due to their wide plateau region in compressive stress–strain curves. Also, porous materials’ energy absorption capability is greater than bulk metals owing to the extensive plateau regime. Thus, there is an increasing necessity to understand and characterize the strain-rate dependant mechanical properties of porous metals. However, most of the literature investigating porous metals’ mechanical properties is usually limited to the quasi-static mechanical properties such as Young’s modulus, compressive/tensile yield or ultimate strengths, etc. A limited investigation has been made to understand the deformation mechanisms, the effect of porosity on the high strain-rate mechanical properties, and the fracture behavior of porous metals subjected to these loading conditions. The mechanical testing and characterizing of materials subjected to high strain rates are complex. Most of the literature discussing the dynamic mechanical properties was directed towards investigating the macroscopic dynamic behavior or the effect of microstructure on the dynamic material properties. Thus, this section will discuss the method for testing, the obtained mechanical properties, their dependence on the material’s porosity, and the fracture behavior of these porous metallic structures subjected to high strain-rate loading [144].

**Figure 14.**
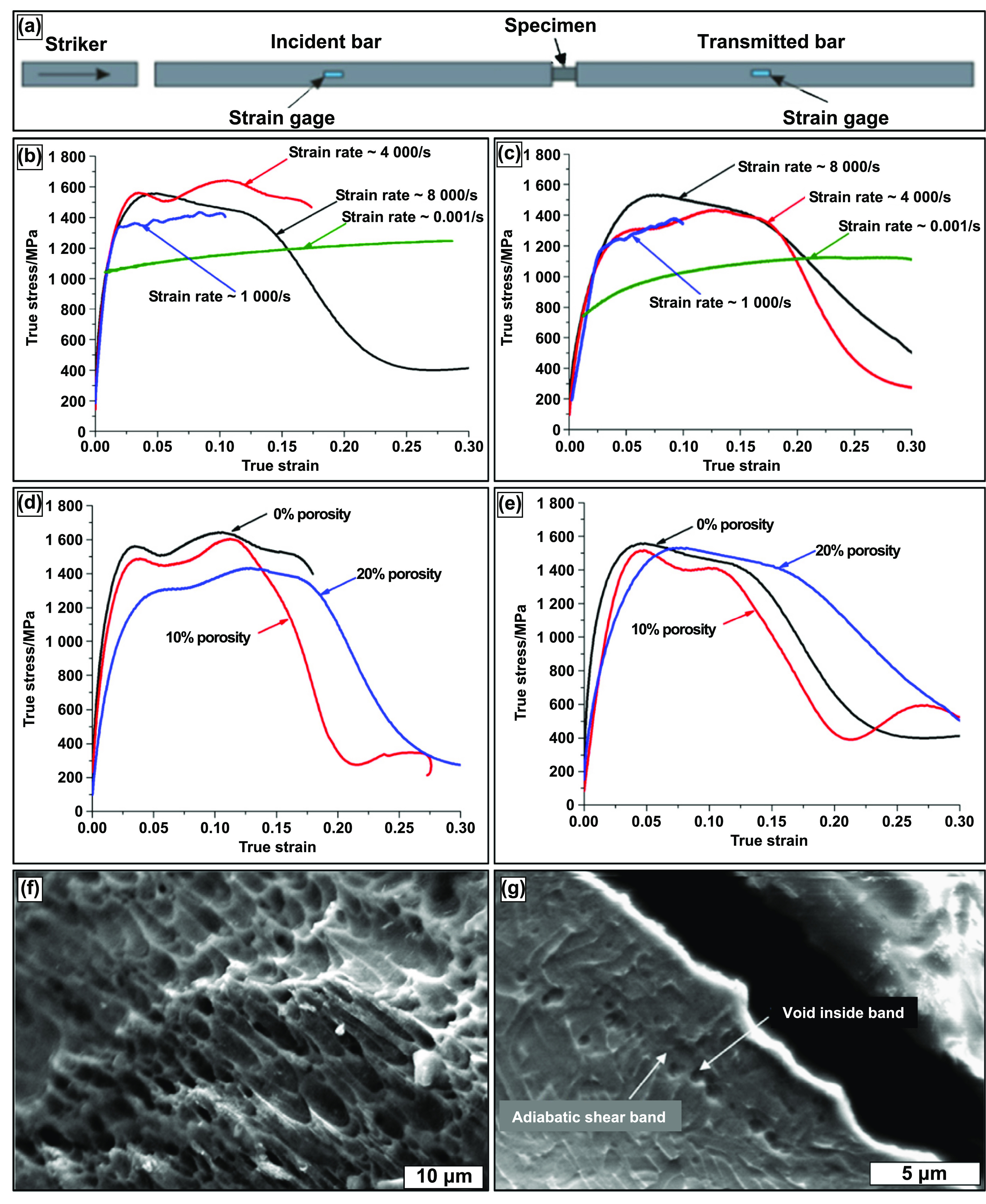
Split Hopkins pressure bar testing and results of dense and porous Ti6Al4V. (a) Schematic of the split Hopkinson pressure bar for high strain rate compression testing. Comparison of true stress–strain plots for (b) 0% porosity and (c) 20% porosity Ti6Al4V LENS samples tested at various strain rates of (d) 4 × 10^3^ s^−1^ and (e) 8 × 10^3^ s^−1^ tested samples of Ti6Al4V of varying porosity. SEM micrographs of the fracture surface for the 20% porosity samples tested at a strain rate of 4 × 10^3^ s^−1^ indicating (f) dimpled structures and (g) shear bands. Reprinted from [145], Copyright (2012), with permission from Elsevier.

In most investigations, the quasi-static and low strain-rate compression tests could be conducted in traditional universal testing machines. However, the most preferred testing method for high strain-rate compression loading is the split Hopkinson pressure bar (SHPB), where the material response to high strain rates in the range of 10^2^–10^4^ s^−1^ could be studied. In this testing method, a cylindrical specimen is to be sandwiched between two long bars, the incident bar, and the transmitted bar. A third bar, the striker, is used to strike the incident bar’s free end, generating a compressive stress pulse that traverses through the incident bar toward the transmitted bar. A schematic of the SHPB method is shown in figure 14(a) [145]. At the interface of the specimen and the incident bar, a part of the compressive stress pulse is reflected as a tensile stress pulse, and the remaining fraction is transmitted through the specimen to the transmitted bar as a compressive stress pulse. The magnitude of these stress pulses is such that the incident and the transmitted bars remain elastic, but the specimen experiences plastic deformation. These reflected and transmitted pulse amplitudes of the corresponding elastic strains are recorded using the strain gauges located on each of the incidents and the transmitted bars and could be used to derive the stress–strain relation for the specimen, given by the following:

\begin{align*}&amp;{\text{engineering strain rate}}:\mathop {{e_s}}\limits^. = - \frac{{2{c_b}}}{{{l_s}}}{\varepsilon _R}\left( t \right),\\ &amp; {\text{engineering}}\,{\text{strain}}:\,{e_s} = \mathop \int \limits_0^t {\dot e_s}dt,\\ &amp; {\text{engineering}}\,{\text{stress}}:\,{s_s}\left( t \right) = \frac{{{E_b}{A_b}}}{{{A_s}}}{\varepsilon _T}\left( t \right),\\[3pt] &amp; {\text{elastic}}\,{\text{wave}}\,{\text{speed}}:\,{c_b} = \sqrt {\frac{{{E_b}}}{{{\rho _b}}}}. \end{align*}

In the above equations, Young’s modulus and the density of the bars are indicated as *E_b_* and *ρ_b_*, the specimen length is given as *l_s_*, and the reflected and the transmitted strain amplitudes measured by the strain gauges are *ϵ_R_* and *ϵ_T_*, respectively [145, 146].

The compressive stress–strain curves for most porous metallic structures depicted increased yield strength and energy absorption capability for high strain-rate loading. Also, the stress–strain curves of most porous metals indicate three different regimes: linear elastic regime, an extended plateau regime indicating nearly constant flow stress, and the final densification regime where the collapsed cells are compacted and lead to a steep rise in the stress before failure. Moreover, most of these investigations have observed that irrespective of the porosity, the material’s yield strength and the extended plateau region tend to increase with the increase in strain rate [144, 145, 147, 148]. This could be observed for the LENS-manufactured Ti6Al4V samples of varying porosities tested at varying strain rates, as shown in figures 14(b) and (c). Also, from figures 14(d) and (e), it could be observed that, as the porosity increases, the ductility tends to increase for the porosity increase in the range of 10%–20%, and the same decreases for the porosities in the range of 0%–10% [145]. The results of this investigation, in comparison to similar investigations on the high strain-rate properties of porous Ti6Al4V, seem to be more or less in agreement with an additional indication of the improved mechanical properties of LENS-prepared porous Ti6Al4V. Some other investigations on porous aluminum and iron also suggest similar trends in mechanical properties with the strain rate [142, 144].

Additionally, the microstructural analysis of the fracture surface of porous samples subjected to high strain rates revealed dimpled structures (figure 14(f)) along the fracture surface, LENS-prepared Ti6Al4V displayed ductile deformation. Furthermore, SEM images of the polished fracture surface also indicated shear bands (figure 14(g)) indicative of localized deformation followed by temperature rise and immediate cooling. Thus, in porous metals subjected to high strain rates, the pores tend to act as the failure initiator at the same time as the failure inhibitor. This implies that the pores act as the nucleation site for forming the adiabatic shear band, leading to failure. However, the role of pores as a failure inhibitor is vaguely understood and primarily attributed to the fabrication process of the porous metal [145].

##### 3.2.3.2. Fatigue properties.

Porous metallic structures are subjected to cycling loading conditions in most applications where porous metallic structures are used. Thereby, aside from the static load-bearing capability of porous metals, it is essential to characterize the cyclic fatigue characteristics. In orthopedic implant structures, during their lifetime, they are mostly subjected to cyclic compression or cycling bending loading. Thus, most of the literature concerning the characterization of fatigue properties of porous metallic structures for orthopedic implant applications is focused on understanding the compression-compression fatigue or rotational bending fatigue characteristics [97].

Fatigue of bulk material is technically defined as the failure of a material or a structure subjected to a cyclic load lower than its ultimate or yield strength. Generally, there are three significant steps in which fatigue failure occurs: crack initiation, crack propagation, and, finally, catastrophic failure. One of the major contributing factors to fatigue failure is the presence of a stress concentration point or region which would act as a crack initiation point. These stress concentration points usually correspond to any flaws or defects that are prevalent on the surface or at the subsurface of the specimen or, in some cases, microstructural features. In the case of porous metallic structures, the pore shape and morphology of the pore distribution act as the source for stress concentration points. Thus, there is an apparent reduction in the fatigue strength of porous metals compared to bulk materials. Moreover, it is crucial to understand and characterize porous metallic structures’ fatigue properties to tailor their porosity and other features to obtain the desired fatigue strength [97, 149].

In the case of conventional processing methods for porous metals, a considerable amount of literature is available, in where, investigation into the fatigue properties of varying porous metals is reported. However, owing to the advantages of using AM-based technologies, particularly in orthopedic applications, there is an immense drive toward understanding the fatigue behavior of porous metals manufactured using these technologies. In most of these studies, the effects of porosity, the process parameters, and the applied stress levels on fatigue properties have been investigated.

According to most literature, compression-compression fatigue testing is conducted on cubic or cylindrical specimens using universal testing machines by applying a sinusoidal compression loading with a frequency of about 15 Hz [97, 149, 150]. The test setup’s force applied to the samples is chosen so that the applied stress levels are between 0.2–0.8 *σ_y_*. In the case of the rotational-bending fatigue testing case, a dog-bone-shaped specimen of standard dimensions is subjected to bending load and rotated at a speed corresponding to the 15 Hz frequency. The rotation of the specimen tends to apply a cyclic, fully reversed tension-compression stress state at any point on the sample surface.

The *S*–*N* curves for the absolute and normalized stress values were reported in an investigation concerning the compression–compression fatigue behavior of porous CpTi with different porosities ranging from 65%–84% [149]. Also, a power law curve was fitted for the normalized stress value *S–N* curve. The absolute stress *S*–*N* curve revealed that the fatigue behavior followed the order of the yield stress of porous CpTi. The fatigue behavior observed from these tests indicated the typical three-stage fatigue failure. Although most combinations of porosity and applied stress level indicated these three stages of fatigue failure, their shape was not similar, and the transition points between stages were hard to detect. The implication of the normalized stress level *S–N* curve, where *S–N* curves for structures with different porosities were reported, that a fitted power law curve could give an empirical model for estimating *S–N* curves for porous structures built using SLM, which could be used as a basis for predicting fatigue properties of porous structures when no experimental *S–N* curve is available. However, investigations of some other porous structures indicated deviation from this model. For instance, a study investigating the fatigue properties of solid Ti6Al4V showed a much higher endurance limit (0.4 *σ_y_*), and another study reported a slightly higher endurance limit (0.15–0.2 *σ_y_*) for porous coated Ti6Al4V compared to the predicted normalized endurance limit of (0.12 *σ_y_*). This deviation from the normalized endurance limit for porous structures is because of either surface roughness-induced notch sensitivity, intrinsic manufacturing limitations such as strut thickness, or the residual thermal stresses induced during the manufacturing processes, such as SLM.

In other investigations, the compression-compression and rotational bending fatigue behavior of porous NiTi alloy fabricated via LENS were studied. In one study, it was observed for 1%, 10%, and 20% porous NiTi alloy samples that these followed similar behavior as the metal foams, i.e. as the amplitude of the cyclic stress applied increased, an increase in the quantity of strain accumulated was observed and as the critical stress amplitude was surpassed a sharp increase in strain accumulation was observed. In the other study, the rotational-bending fatigue life of 0% and 10% porous NiTi alloy samples was investigated, and it was observed that the 10% porous sample showed about 54% reduction in fatigue stress [151].

**Figure 15.**
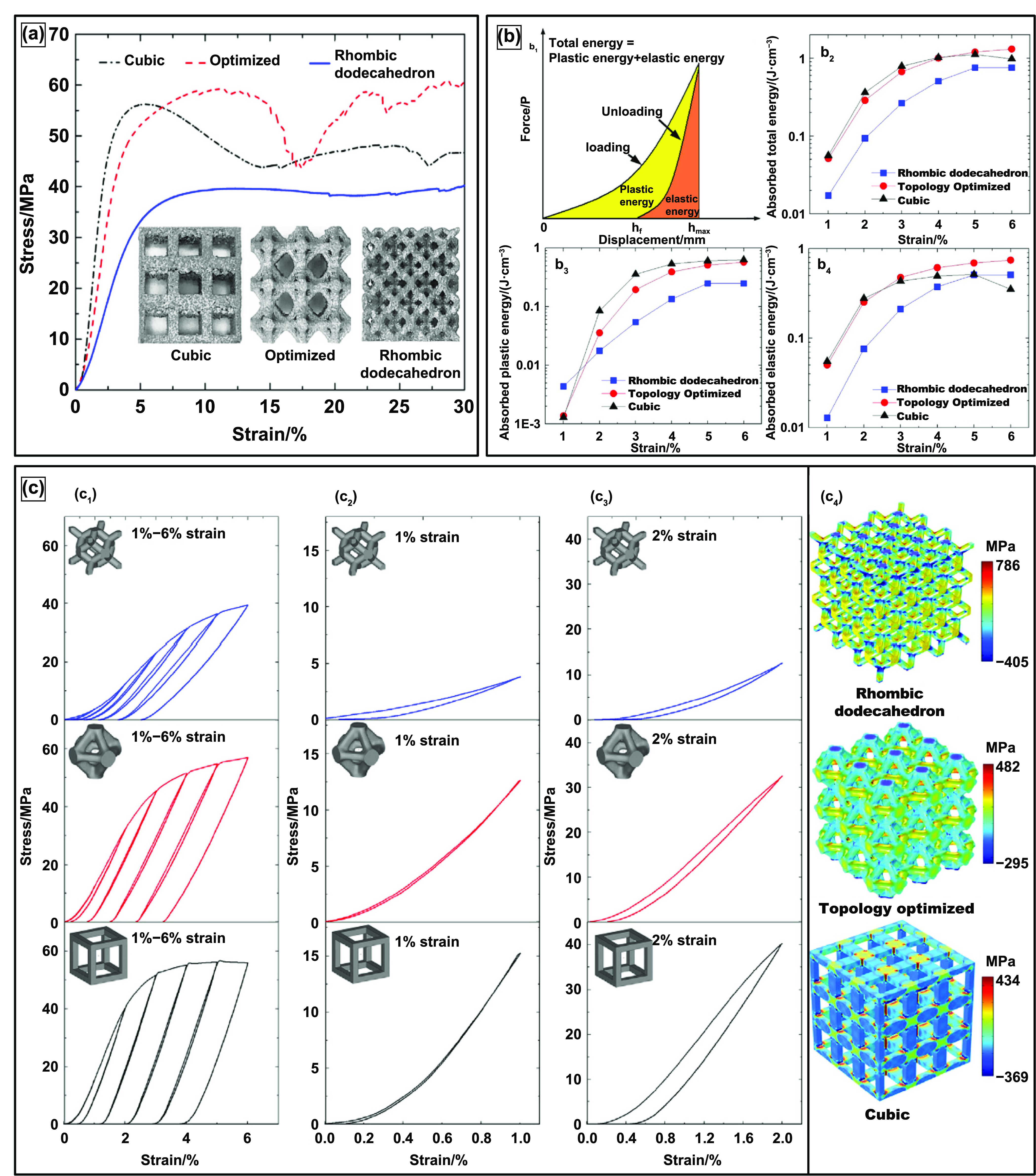
Plastic deformation behavior and energy absorption for porous β-type biomedical titanium. (a) Displays the compressive stress–strain curves for the SLM-produced lattices, (b) displays the (b_1_) diagrammatic sketch of energy absorption for the three lattices, specifically, (b_2_) the total energy absorption, (b_3_) the plastic energy absorption and (b_4_) the elastic energy absorption domain. (c) Displayed are the stress–strain curves for cyclic loading of the three lattices at (c_1_) 1%–6%, (c_2_) 1%, and (c_3_) 2% strain level, as well as (c_4_) the FEM results for the three lattices at 1% strain level. Reprinted from [152], Copyright (2018), with permission from Elsevier.

As previously mentioned, *β*-Ti alloys have also been investigated for biomaterials in bone implant repair due to their reduced Young’s modulus—which is better suited for hard tissue replacement [152]. Much research has addressed the mismatch in modulus by tailoring and implementing a suitable geometry to lower the effective modulus of the implanted material to that of the host hard tissue, figure 15. Energy absorption is a critical material property which is affected drastically by the lattice structure; this material property recieves significant scrutiny across many industries. In figure 15, three SLM-printed lattices’ energy absorption mechanisms were investigated. A depth into such mechanisms, such as their plasticity mechanism and local stress concentration, was conducted for each lattice at differing strain levels. The investigation balanced the bending and buckling stress maximizing energy absorption, displaying a low Young’s modulus of ∼2.3 GPa and an ideal compressive strength of ∼58 MPa [152]. The work by Liu *et al* was conducted using the *β*-type Ti24Nb4Zr8Sn alloy; this alloy is desirable for its non-toxic constituents, low Young’s modulus, and high strength [153]. The Ti24Nb4Zr8Sn alloy exhibited an increase in both elastic and plastic energy absorption. The plastic energy absorption directly resulted from dislocations and slip bands forming and moving within the structural struts. It was established that the total energy absorbed could vary with the shape of the lattice structure. The geometrical differences in the strut matrix alter the stress distribution, local stress concentration, and dislocation motion—which are the direct mechanism for energy absorption in lattice structures. Although *β*-Ti alloys are still being researched, their poor fatigue resistance than Ti6Al4V has yet to be resolved in most compositions.

### 3.3. Electrochemical testing and properties

Often novel literature reports a material under investigation meeting or exceeding the requirements for matching modulus, reducing stress shielding, reducing wear debris, and reducing released toxic ions. Even *in vitro* studies can result in favorable results, such as antibacterial properties or improved cell attachment and proliferation. All great attributes for a material to exhibit when considering biomedical implants, although none give us an insight into the corrosion behavior of the material *in vivo*. Most commonly, corrosion in the form of oxidation is favorable in materials that will see use in the aerospace industry or the biomedical device industry; that being said, it is controlled and results in a dense, coherent passivation layer that protects the bulk material from further side reactions when in use. If a passivation layer is non-existent, the material may still be susceptible to react with oxygen or like constituents within the body, for example, in the biomedical device industry. For this reason, science has coupled electroanalytical techniques, *in vitro* and tribological testing for an in-depth corrosion-based understanding of the material; to accomplish such testing, most commonly, a corrosion cell is modified by using simulated body fluid and implementation of a tribological device.

#### 3.3.1. Corrosion apparatus.

**Figure 16.**
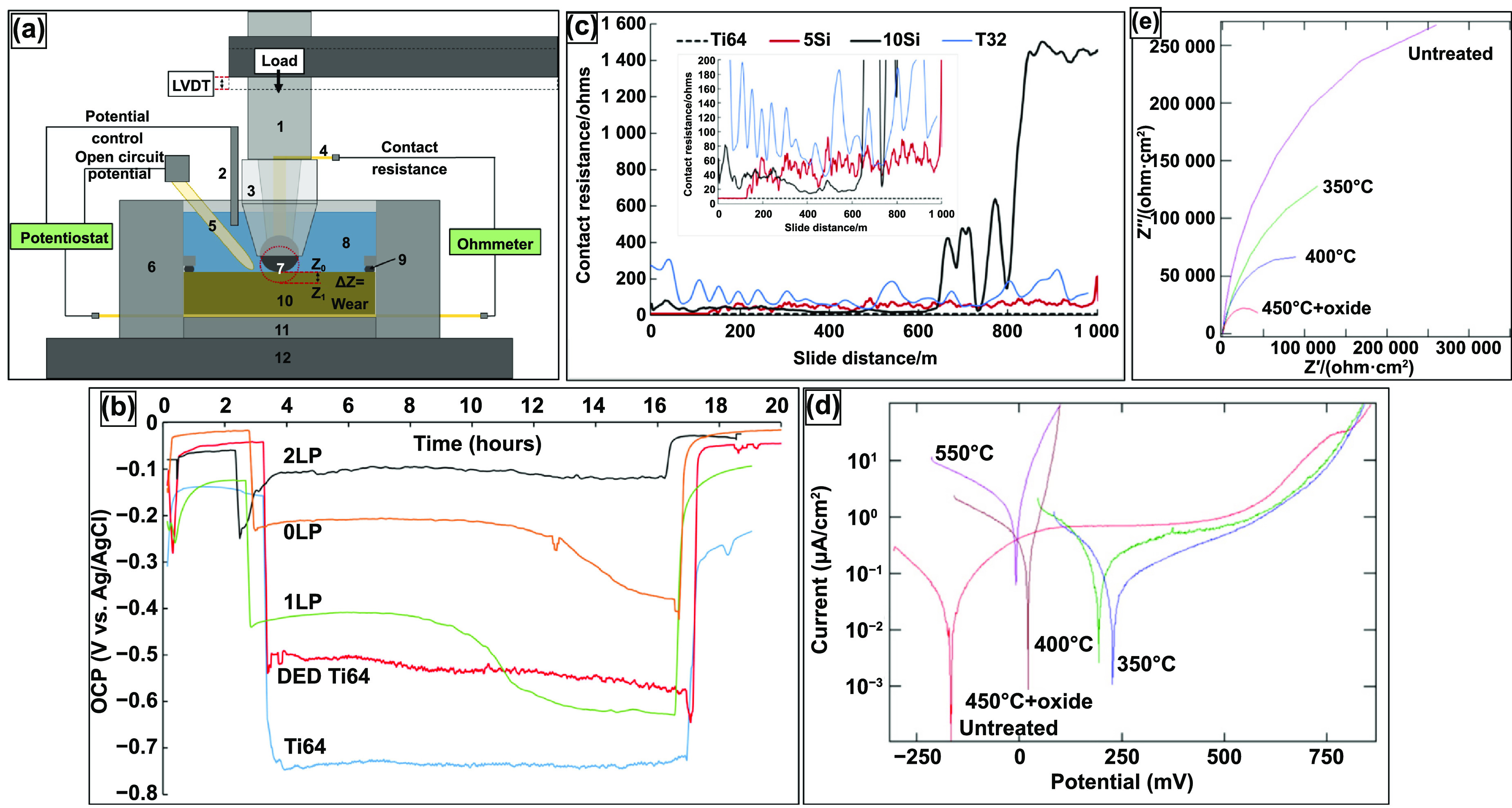
Schematic representation of (a) tribocorrosion-cell setup, displaying the data acquisition unit for open-circuit potential (OCP) (potentiostat) and contact resistance (CR). Additionally, displayed are (1) PEEK sample holder rod, (2) counter electrode, (3) PEEK sample holder cap, (4) conducting terminal end, (5) reference electrode, (6) media bath, (7) counter wear ball/pin, (8) electrolyte media, (9) media bath seal, (10) the working electrode (analyte) with the ability to translate or rotate, (11) analyte holder, (12) stage and (13) loading arm. Reprinted from [155], Copyright (2020), with permission from Elsevier. (b) Open-circuit potential curves under idle, tribological wear, and once recovery (re-passivation) domain. Reproduced from [154], with permission from Springer Nature. (c) Contact resistance curves were attained through the use of an in-line two-wire ohm meter, with the use of a conductive wear ball. Reprinted from [155], Copyright (2020), with permission from Elsevier. (d) Potentiodynamic polarization curves in Ringer solution for CoCr produced via varying processing temperatures. (e) Nyquist plots for CoCr produced via varying processing temperatures after 2 h immersion in Ringer solution. Reprinted from [158], Copyright (2011), with permission from Elsevier.

A typical corrosion-cell setup requires media (electrolyte), a working electrode (WE), a counter electrode (CE), a potentiostat/galvanostat, and a reference electrode (RE). The specimen acts as the WE for the electroanalytical testing of metals, and platinum or stainless steel is most commonly the CE material of choice. A typical setup is displayed in figure 16(a), in which the loading arm [6] may be added for additional *in situ* tribological testing. Two forms of material electroanalytical testing exist, passive and active, testing. The loading arm and the CE are omitted for passive testing, as no electrical driving force is applied. In active testing, a potential is applied through the CE and WE. The applied potential is measured to the RE. In active testing, all three electrodes require submersion in the electrolyte; data logging for electroanalytical value reporting is done using a potentiate.

#### 3.3.2. Passive and active testing.

Passive testing, inherently, is an open circuit with no load and no power source submersed in the electrolyte or in line with the WE circuit. The testing is typically used to acquire the resting potential, equilibrium potential, free corrosion potential, or open circuit potential (OCP)—all of which can be used synonymously—of a material in an electrolyte of choice to a standard RE. The testing is under idle conditions, so characterizing reaction dynamics and kinetics is not within the scope of the test, but the observable stability of passivation formation and its stability at idle conditions is. The OCP is measured with the RE and is the observable difference in potential between the RE and WE. The OCP measurement coupled with tribological testing gives rise to the OCP curves displayed in figure 16(b). The sample (WE) is submersed in the cell media and allowed to reach equilibrium; once equilibrium is attained, tribological testing can commence. The testing will run for a standardized amount of time for all specimens, and then the loading arm is removed, and the worn surface is continually monitored by OCP acquisition. In this form of testing, a material’s electrochemical performance is monitored under mechanically idle conditions and mechanical loading and wear. A generally accepted shift in OCP from control to treated samples is ideal when positive. A positive shift in OCP comes hand-in-hand with a decreased tendency for corrosion to occur. Afrouzian *et al* concluded that silica introduction into Ti6Al4V allowed for a positive or noble shift in OCP under idle and tribological loading conditions [154]. It was further shown that laser treatment of the depositions allowed for the further reaction of silica with Ti and stabilization of refractory phases resulting in the material’s improved OCP performance. Avila *et al* concluded similar results with silicon introduction into Ti6Al4V by acquiring contact resistance (CR) values. *In situ* formed silicide presence on the worn surface accounted for increased CR values after the initial running-in wear regime associated with tribological testing [155]. Additionally, it was reported that the silicide-based film reduced the worn volume loss—a decrease from 1.61 × 10^−9^–4.86 × 10^−10^m^3^. Passive testing not only allows for characterizing a material’s tendency to corrode but the testing can also be coupled with tribological testing to observe a material’s performance in corrosive media under mechanical wear. On the other hand, active testing deviates from passive testing as potential is applied.

Active testing is performed by application of a varying voltage; a voltage sweep from a negative potential to a positive potential, straddling the breakdown potential, as seen in figure 16(d). The testing is known as potentiodynamic polarization and measures current through the WE and CE; both corrosion potential (*E*_Corr_)—synonymous to breakdown potential—and corrosion current (*I*_Corr_) may be determined from such testing. The determination of *E*_Corr_ and *I*_Corr_ is done by Tafel extrapolation of the potentiodynamic curve when the shift in anodic current to cathodic current occurs. Additionally, corrosion rates, passivation domain, and electrolyte breakdown potential may be determined. Lutz *et al* determined that heat treatment of CoCr along with nitrogen plasma immersion and ion implantation results in an increased current requirement and a noble shift, *I*_Corr_ and *E*_Corr_, respectively, thus representing the increased electrochemical stability of CoCr after treatment. It is essential to realize that these active tests drive the redox reaction to occur under somewhat undesirable or steady-state terms. Most commonly in the body, the applied potential used in these active tests is not seen in physiological conditions but is used and applied to accelerate the testing. It is known that porous metals exhibit increased degradation *in vitro* and *in vivo* due to their increased surface area exposed to a media allowing for increased surface reaction. The material degradation occurs with cyclical surface passivation and passivation layer spallation or dissociation into the media; in this case, the media would typically be an electrolyte. A porous structural lattice’s added porosity and complex shape only increase the surface area/media exposure. Therefore, applying a constant potential and measuring the current density for a metal derivation of the corrosion rate may be determined—typically in units of millimeters per year (mmpy). Even porous metals can be categorized this way when considering their degradation over time. A subsequent testing method that should follow an active test, such as potentiodynamic polarization or current density determination, is electrochemical impedance spectroscopy (EIS). The results of such testing indicate the material’s intrinsic chemical behavior and, therefore, would not depend so much on the structure’s geometry.

EIS is a compelling testing technique that gives insight into a material’s resistance to reaction at the interfacial surface of the material and electrolyte [156]. Testing is conducted by applying an alternating current (AC) and measuring the impedance when varying the frequency. Plots associated with this testing are known as Nyquist plots, as displayed in figure 16(e). EIS results allow for the interpretation of the passivation layer as a monolayer or bilayer as well as a constituent presence within the passivation layer. A higher impedance value indicates the surface’s greater resistance to further reaction [157]. J. Lutz discovered that with increased heat treatment temperatures, the corrosion resistance decreased for the CoCr alloy; this is seen as a reduction in impedance with the treated CoCr alloy displayed in figure 16(e). It goes to show that not just when the test suffices in electrochemical behavior of a material. Additionally, most of the observed results from such electroanalytical tests are intrinsic to a material, and surface porosity will not change the values such as *E*_Corr_ or impedance but will so when normalized over the surface area such as current density. Nevertheless, such testing allows for a ballpark range of material development that will be adequate for *in vivo* use; to better tailor materials for *in vivo* use, biological testing should never be substituted.

## 4. Biological properties

A commercial orthopedic implant must be biocompatible and free of toxic compounds. Toxicity is related to the amount of toxin that is present in a substance [159, 160]. Metal ion toxicity comes from most heavy metals, which can pose a severe health hazard to organs and cells [161]. However, many metal ions are also essential minerals to the human body, including barium (Ba), chromium (Cr), iron (Fe), and selenium (Se). Excessive doses of these essential minerals, on the other hand, can be harmful [162]. Metal compounds also pose a health risk since they are susceptible to chemical breakdown in physiological fluid, called hydrolysis. Such compounds may break down into hazardous chemicals or generate insoluble residuals after a chemical reaction [163]. Excellent biocompatibility with living cells or tissues needed for wound healing, repair, and tissue integration results from the complex interactions between a material surface and its biological host [164]. Any metallic, polymeric, or ceramic implant must show excellent biocompatibility, allowing cells and tissues to function and serve as a template for osteogenesis and angiogenesis for bone repair, creation, remodeling, and healing [164–166].

### 4.1. Biomechanical properties

Exceptional mechanical performance including fatigue and corrosion resistance make Ti and its alloys favorable to use in load-bearing applications [167]. High-strength implants help patients participate in physical activities while protecting them from undesirable fractures [168]. Stress-shielding can be avoided with implants having a suitable elastic modulus. Stress shielding occurs when the implant takes up physical stresses rather than the bone due to the mismatch between its elastic moduli and surrounding bone. Stress shielding causes bone shrinkage, implant loosening, and even failure. The elastic modulus of cancellous bone ranges from 1–15 GPa, but the cortical bone has a substantially higher elastic modulus, ranging from 10 to 30 GPa [119, 169]. The elastic modulus of metal implants should be similar to the native bone to minimize stress shielding. However, it is usually higher than bone; for example, the elastic modulus of commercially pure CpTi and Ti6Al4V is around 110 GPa, substantially higher than that of cortical bone. As a result, implant design must reduce the elastic modulus to minimize stress shielding. Open-cell porous metal implants via AM can meet such requirements of low elastic modulus allowing new bone tissue ingrowth and vascularization. However, there are still questions related to long-term implant stability since inert metal implants do not biodegrade or bioresorb over time; they remain as foreign bodies in the patient from a physiological point of view. This, frequently result in adverse reactions from the body toward the inert metal, such as discomfort, microbial infection, inflammation, and extended systemic dysfunction from leaching, which hugely impacts the patient’s quality of life [170]. Implants composed of non-biodegradable materials such as titanium alloys and stainless steel can sometimes stop bones from growing normally, necessitating subsequent surgery to encourage further bone growth. As a result, developing porous biodegradable metals for an implant is desirable.

### 4.2. Importance of porosity in implant applications

**Figure 17.**
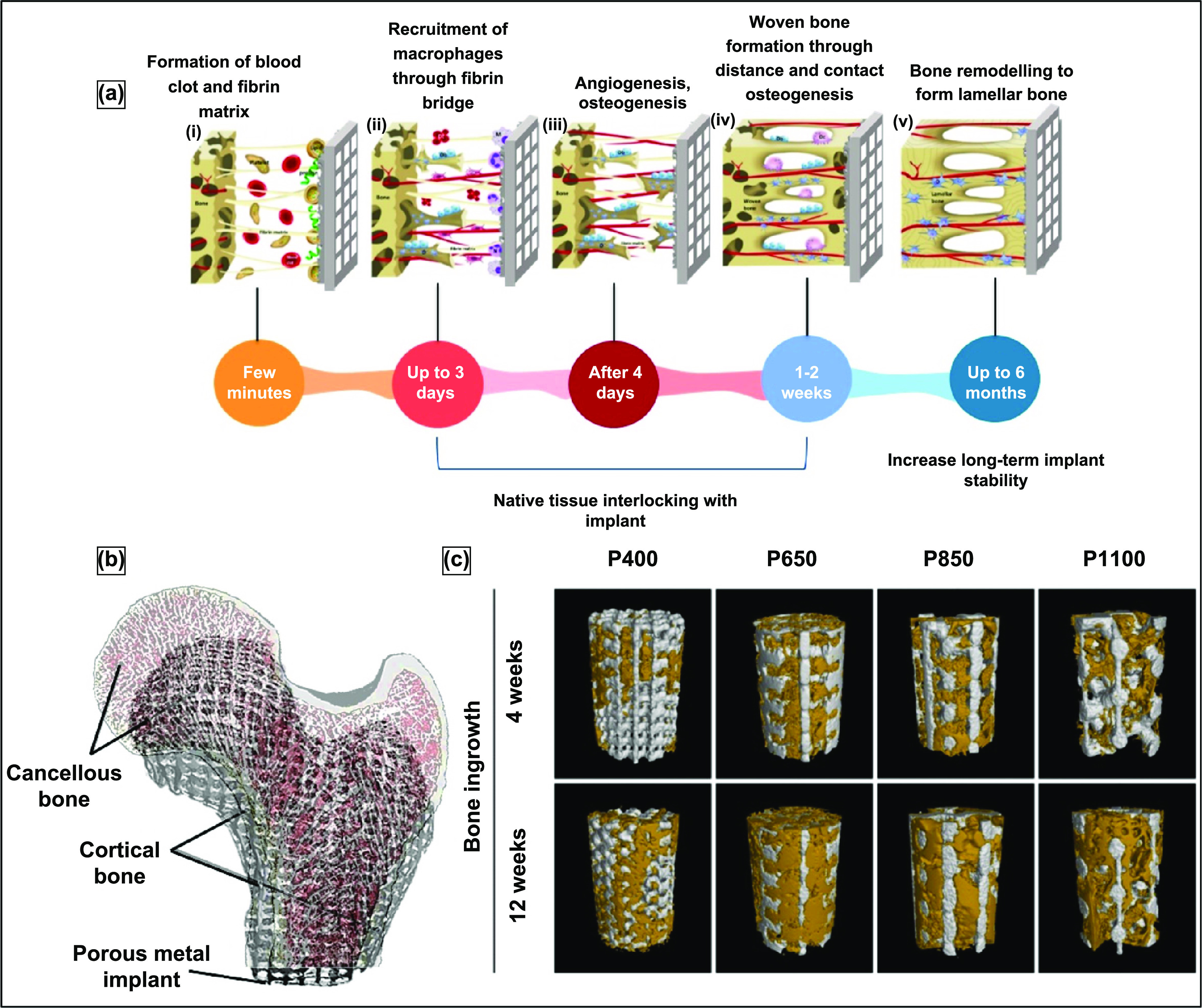
Importance of porosity in implant applications, (a) Schematic of *in vivo* osseointegration in porous metallic implants in stages of (i) blood clot formation at the defect site, (ii) osteoclast and macrophage recruitment in the resorption stage, (iii) pore facilitated neovasculature and osteoblastogenesis in the reversal phase, (iv) woven bone formation and (v) complete, mature bone ingrowth in pores for long-term fixation [174]. John Wiley & Sons. © 2019 The Authors. Journal of Biomedical Materials Research Part A published by Wiley Periodicals, Inc. (b) Represents stacked images of the anatomical femoral head and a digital model of an additive-designed porous titanium femoral bone showing the precision of AM techniques toward replacement of impaired bone; (c) Actual micro-CT based reconstructed images of bone growth in porous titanium implants in rabbits after 4 and 12 weeks of femur condylar implantation. Reprinted from [190], Copyright (2019), with permission from Elsevier.

Cortical and cancellous bones are porous, open-cell composite substances put down by osteoblast (OB) cells. The cortical bone is the hard outer shell having reduced porosity, and the cancellous bone is a highly porous structure at the inner part of the bone [171]. Cancellous bone porosity can vary between 30% and 95%, having pore sizes between 100 and 1000 *μ*m [171–173]. Since porous scaffolds can support tissue ingrowth from the native bone as well as sustain the homeostatic tissue performance ultimately leading to the formation of a new bone microarchitecture whether woven bone or lamellar bone depending on the metallic implant’s characteristics and pore properties. Therefore, there has been a significant interest in designing and manufacturing those structures in recent years, figure 17(a) [174].

Biomedical implants and devices with high degrees of porosity are of interest because of their excellent osseointegration abilities [175, 176]. An ideal porous implant should have interconnected open-cell pores for neo-vasculature [177]. A minimum pore size of 100 *μ*m is necessary for cells to migrate and be transported for osteogenesis [177]; ideally, a 200–350 *μ*m diameter pore size is preferred [171]. Torres-Sanchez *et al* [178] investigated the biological properties of porous Ti scaffolds with four distinct pore sizes: 45–106 *μ*m, 106–212 *μ*m, 212–300 *μ*m, and 312–300 *μ*m. The Ti scaffolds were tested *in vitro* with osteosarcoma-OBs and cultured for 12 d to assess cell adhesion and proliferation. It was reported that cell adhesion is enhanced by small pores, while large pores aid cell multiplication. It has been reported that having a larger pore size distribution with macro-pores (>50 *μ*ms) and micro-pores (<20 *μ*ms) are better for scaffold design [89, 179, 180]. While macro-porosity helps in cell-materials interaction and osseointegration, micro-porosity helps with osteogenic protein adsorption.

For the past two decades, applications of 3D Printing have been widespread in the biomedical sector—from surgical models to prosthetic design and fabrication. Polymer-based 3D Printing approaches, such as fused deposition modeling, and stereolithography, are examples of techniques used to build molds to cast predesigned 3D structures [179, 181, 182]. Predesigned tailored prostheses based on patients’ imaging data are ideal for patient-matched devices [180, 183]. An example of wrist and knee prostheses via 3D Printing is given in figure 17(b), with reported favorable outcomes [184]. Designing and manufacturing complex implant structures used to be complicated; however, those can now be easily met with 3D Printing technology based on CAD. The capacity to produce interconnected porous constructions with predictable and specified unit cells is the fundamental advantage of AM technology over traditional approaches [185–187]. Pore size, shape, and porosity are all predetermined, and pore morphology displays a systematic pattern rather than a random distribution [188, 189]. Influencing cell behavior and impacting tissue regeneration in pores, porosity parameters, and scaffold features are essential to bone ingrowth [190]. Furthermore, because porosity affects implants’ compressive strength and elastic modulus, their mechanical properties can be tweaked to mimic natural bone ones. Thus, by precisely managing implant porosity, stress-shielding effects after implantation could be minimized or prevented [191].

### 4.3. Pore characteristics influencing bone ingrowth

#### 4.3.1. Pore size.

**Figure 18.**
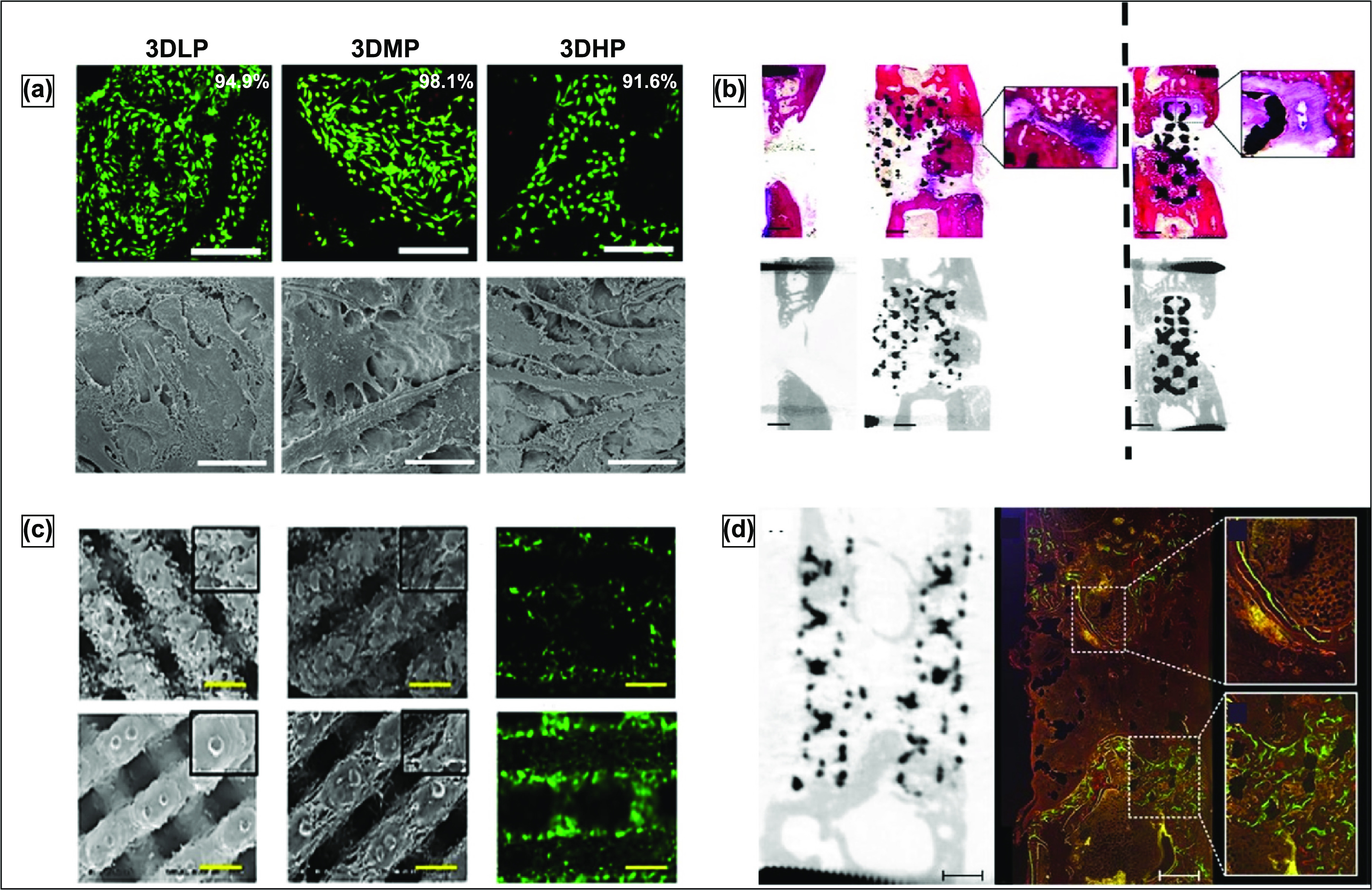
Contribution of total porosity in metallic implants toward osseointegration, (a) live/dead analysis of MG63 osteoblast-like cells on porous Ti6Al4V constructs with porosity <20% (3DLP), <50% (3DMP), and >70% (3DHP) showing highest live cell confluence ∼98% for 3DMP indicating volume fraction porosity plays an integral part in cellular viability and proliferation irrespective of pore size. Reproduced from [197]. © IOP Publishing Ltd CC BY 3.0. (b) (left panel) Shows osseointegration in porous Ti scaffolds with 88% porosity where the woven bone was not able to fully bridge the implant in the intramedullary *in vivo* model and focally developed at the periphery of the implants. This is also called hypertrophic non-union as compared to the (right panel) implants with 68% porosity reporting a higher amount of pore bridging of the woven bone with a fibrocartilage development at the periphery [198] John Wiley & Sons. Copyright © 2012 Orthopaedic Research Society; (c) 70% porous Ta (top) and 70% Ti (bottom) scaffolds showing a difference in bone marrow-derived mesenchymal stem cell growth as a function of surface roughness of two metals as a comparison. Reprinted from [199], Copyright (2019), with permission from Elsevier. (d) Further fluorochrome labeling of 88% porous Ti scaffolds showing the limited progression of pore bridging even up to 12-weeks post-implantation [198]. John Wiley & Sons. Copyright © 2012 Orthopaedic Research Society.

Pores can form because of inter-particle bonding, resulting from incomplete melting of metal powders. Pores can also be pre-designed in CAD models [91]. The pores addressed in this article are primarily pre-designed types. Such surface pores are critical for osteointegration and bone growth, figure 18 [19, 192–194]. Determining what pore size and distribution is best for bone ingrowth is crucial. Pore size is vital in implant-induced osteogenesis because it allows OBs and mesenchymal cells to migrate and proliferate [195]. Nutrient supplementation and cell attachment to the scaffold should be possible with the optimum pore size [196].

One hundred-micron pore size is the minimum requirement for an implant for preferable bone ingrowth *in vivo* [171]. As a result, implants made using AM often have pore sizes above 100 *μ*m. The effects of a series of porous scaffolds with pore diameters of 177 *μ*m, 383 *μ*m, and 653 *μ*m on cell behavior were investigated in one study [197]. With increasing pore size, OB maturation was preferable over proliferation evident from dominant osteocalcin and BMP-2 signatures compared to alkaline phosphatase. *In vitro* studies comparing 640 *μ*m and 1200 *μ*m pore diameters reported higher alkaline phosphatase and osteocalcin levels [93]. Calcium deposition was also higher in the small pore size group, suggesting a more calcium-containing mineralized matrix and better osteogenesis. Pore diameters of 500, 700, and 1000 *μ*m showed a similar tendency, with the 500 *μ*m group having the most potent osteogenic activity [200]. Based on the initial findings, we believe that a pore size of 300–600 *μ*m is ideal. However, merely assessing the extent of osseointegration or cell proliferation is insufficient. Smaller pore sizes might hinder or block metabolite and oxygen diffusion into the center of the scaffolds resulting in an interior necrotic environment and poor implant stabilization. Pore size that is too big is linked to a low bone ingrowth ratio. As a result, if the same amount of bone is created, it is evident that scaffolds with a larger pore size compared to small pores [201]. The ideal pore size for tissue integration is still an active research question.

#### 4.3.2. Volume fraction porosity.

**Figure 19.**
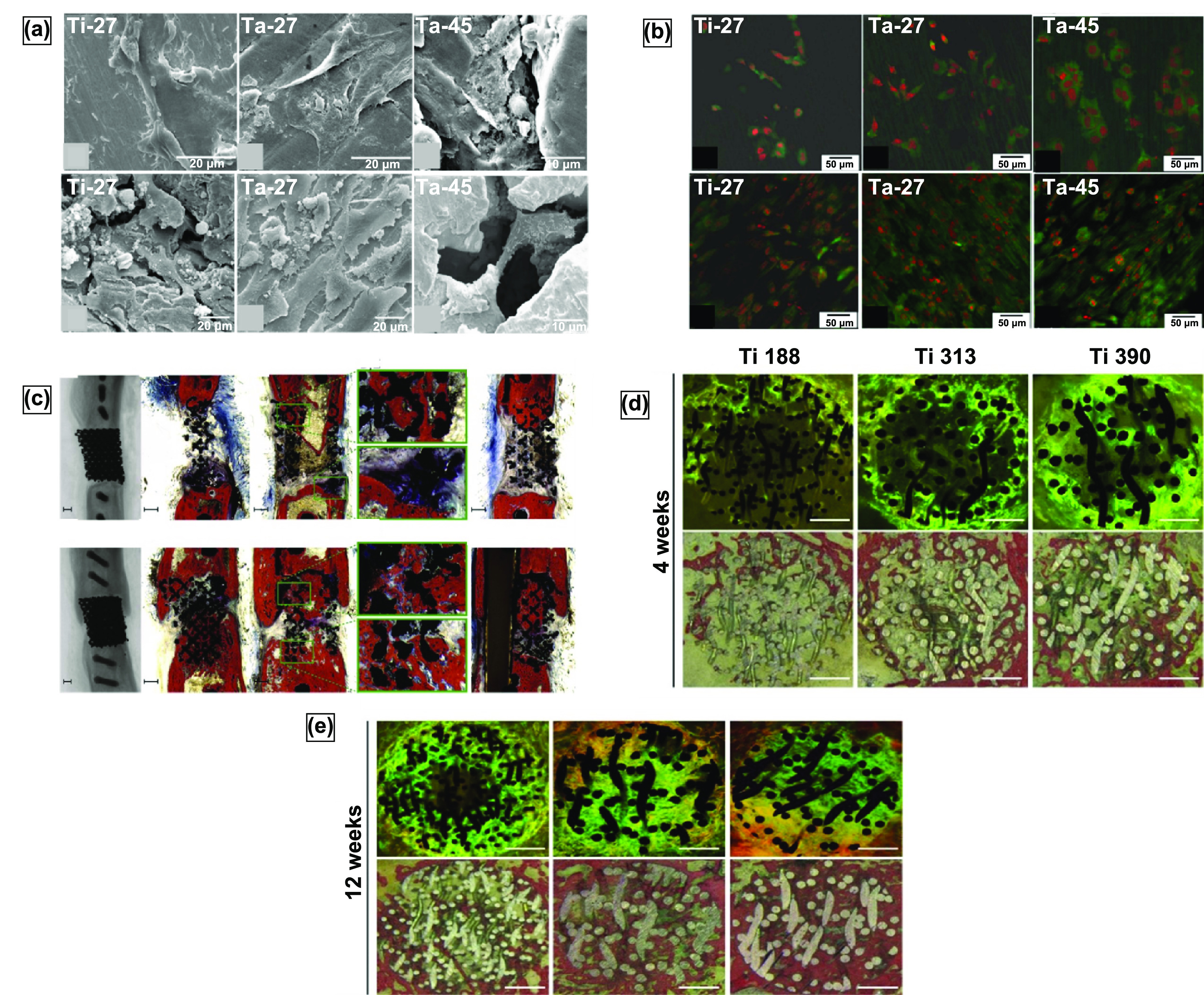
Influence of pore size on osseointegration of porous metal implants, (a), (b) porous Ta implants with 27% and 55% volume fraction porosities were reported to exhibit better cell-material interactions and osteoblast growth/proliferation as well as early-stage biological fixation via morphological assessment through SEM and live/dead confocal assay. Reprinted from [19], Copyright (2010), with permission from Elsevier. (c) Porous Ta implants with overall 80% porosity shows prominent bone ingrowth and full defect bridging 12 weeks post implantation from histological analysis, and high strength interfacial bone-implant bonding from torsion tests on explanted cadaver bone. Reprinted from [192], Copyright (2015), with permission from Elsevier. (d), (e) 70% porous titanium implants fabricated using vacuum diffusion process with hybrid pore sizes shows from 4 and 12 weeks *in vivo* analysis, call differentiation is mainly dependent on porosity and time of healing evident from solid titanium plate had higher differentiated cells than porous titanium while cell proliferation and bone ingrowth is biased upon increasing pore size. Reprinted from [194], Copyright (2016), with permission from Elsevier.

Pore size, wall thickness, and pore morphology influence porous material properties. Porous implants’ porosity with traditional procedures ranges from 30% to 60%. Bone ingrowth is believed to be aided by increased porosity. High implant porosity has been achieved using traditional manufacturing techniques [202]. Increased porosity increases the implant’s surface area, allowing for more cell-materials interactions. A higher porosity suggests a rougher surface, which is favorable for achieving a better interface-locking effect in the early stages of implantation, figure 19 [42, 197–199]. On porous scaffolds, cells attach better. Cells in porous scaffolds express more alkaline phosphatase than cells in non-porous scaffolds, showing that osteogenic differentiation is affected by pore geometry [19]. Human trabecular bone has a 70%–90% porosity, which may be ideal for porous scaffolds. Increased porosity has been shown to contribute to better bone ingrowth in scaffolds with less than 70% porosity [203]. 3D Printed porous Ta implants having an average of 80% porosity showed good bone-implant bonding [196]. In another study, trabecular bone was used as a template to process scaffolds with varying porosities (15%, 38%, and 70%) and shown that the 70% porosity structure was better at stimulating OB differentiation than the low-porosity scaffolds [79]. Similar results with 49%–77% scaffolds porosities showed better bone ingrowth and cell survival [200, 204]. For scaffolds with porosities between 70% and 90%, 80% porosity performed better than 75% in an *in vivo* model during push-out tests and histological analyses [79]. Interestingly, better alkaline phosphatase activity and osteogenic gene expression in scaffolds with a 73% or 81% porosity showed that low porosity was favorable for OB differentiation [79]. It is important to note that higher porosity decreases mechanical strength, and high-porosity scaffolds are unsuitable for any load-bearing applications except for coatings. The interconnectivity among the pores is essential for tissue integration, starting at the surface and going into the center. Closed pores do not support osteogenesis and angiogenesis. Finally, materials with narrow pore throats and a higher detour index show poor osseointegration at the same volume porosity [205].

#### 4.3.3. Pore structure.

**Figure 20.**
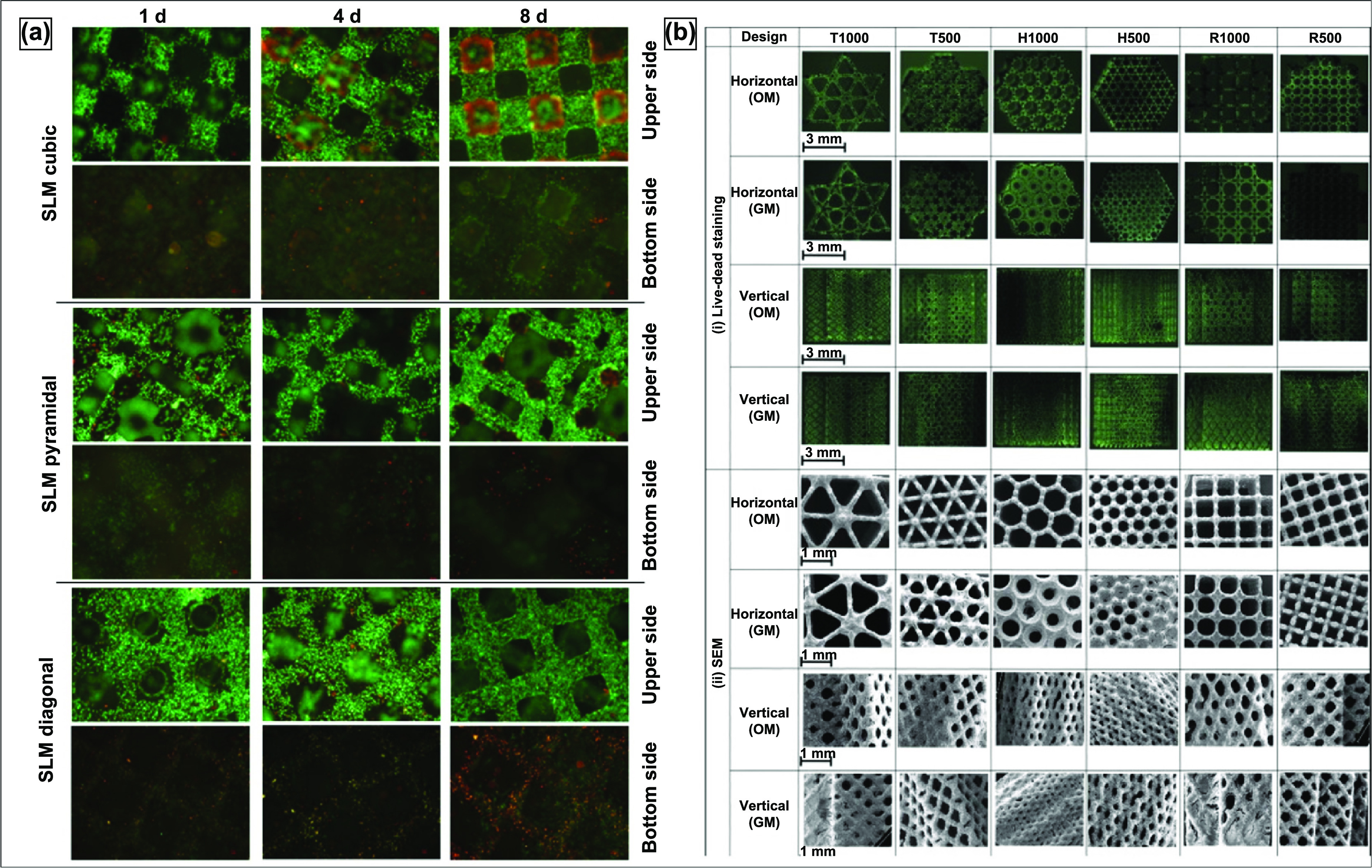
Influence of pore shape on overall cell-material interaction, (a) Osteoblast cultured in a dynamic system on SLM fabricated open porous Ti scaffolds show cell migration as well as enhanced viability for pyramidal pore structure due to enhanced metabolic activity through the open pores such as gas/metabolite exchange compared to cubic and diagonal pore structures which partially hinders the supply of oxygen and nutrients. Reproduced with permission from [200]. (b) Computer design images of live/dead staining and SEM micrographs in the *x–y* and *z*-plane of human periosteum derived cells (hPDC) on six porous Ti6Al4V scaffold designs cultured in osteogenic media (OM) and growth media (GM) for 14 d. SEM images revealed pore occlusion differences between the different designs and culture media. Green fluorescence indicates living cells. Reprinted from [208], Copyright (2012), with permission from Elsevier.

The structure of pores is critical to how cells adhere to the pore walls or struts for regular cellular activities. To better understand the influence of pore structures on cellular activities, the processing of tailored porosity structures is essential. Before the advent of 3D Printing technologies, it was challenging to process controlled porosity scaffolds to study bone tissue ingrowth in porous scaffolds at the micro-scale. Today, typical micro characteristics and pore structures can be obtained using 3D Printing. A CAD design can develop scaffold models with various pore configurations, and 3D porous scaffolds can be manufactured from those predesigned models. Honeycomb-like and cubic structures are two commonly used pore architectures. Honeycombs are designed from a diamond lattice with each strut surrounded by four tetrahedral struts [206, 207]. The honeycomb is fabricated by changing the scanning direction by 90° [93]. Singular pore-structure is most commonly researched in terms of bone ingrowth and not a lot of studies look at different pore morphologies. Biemond *et al* [204] showed wave structure had a higher friction coefficient between wave and cubic morphologies. The high coefficient of friction aids initial interlocking, resulting in better bone formation and initial biomechanics [204]. Cubic, pyramidal, and diagonal pore morphologies were evaluated in another study toward bone integration, findings suggest maximal cell viability and integration for the pyramidal morphologies figure 20(a) [200]. 3D-printed femoral head similar implants with random pore sizes and pore structures showed good cell survival, proliferation, and maturation figure 20(b) [208]. Such results highlight the unique behavior of irregular pores in local growth factor syntheses, for example, bone morphogenetic protein-2 and vascular endothelial growth factor, and promoting bone formation.

### 4.4. Bone induction in a porous metal compound system

**Figure 21.**
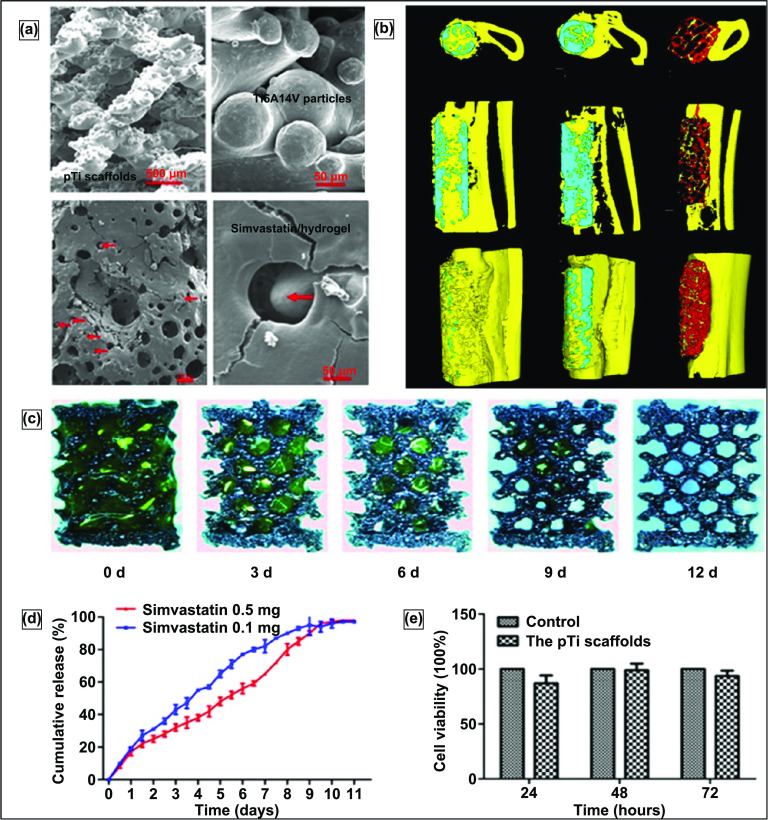
Bone induction in porous Ti scaffold incorporated with simvastatin/poloxamer 407 thermosensitive hydrogel (a) towards enhanced *in vivo* osseointegration evidenced from micro-CT images of rat tibial defect model. Reproduced from [214]. © IOP Publishing Ltd All rights reserved. (b) The higher correlation between neovascularization. Reproduced from [212], with permission from Springer Nature. and (c) new bone volume is observed as a function of simvastatin release (d), which in turn influences cellular viability (e); micro-CT images of enhanced bone formation and porous ingrowth in RGD peptide-modified porous Ta implants at 16 weeks post-surgery in a midshaft segmental defect in New Zealand Rabbits at the proper radius. Reproduced from [214]. © IOP Publishing Ltd. All rights reserved.

Several approaches have been documented for manufacturing porous metal structures for bone formation with the help of cellular queues and polypeptides. Human bone marrow stromal cells (hBMSCs) were the first set of cellular therapies that were used for bone regeneration of allografts. Comparison between porous Ta implants with hBMSCs with natural allografts and allografts supplemented with hBMSCs showed that porous Ta could enhance osteogenic differentiation with hBMSCs equivalent to that of allografts [209]. It has been shown that recombinant human bone morphogenetic protein (rhBMP) can speed up the healing process of the implanted region or decrease adverse interactions between the implant and the surrounding tissues. BMP-7-modified porous Ti aided in subchondral bone regeneration and the formation of new osteochondral tissue and bone. Gelatin-covered Ti6Al4V surfaces [210] where the polyamine layer inspired by adhesive properties of mussels lead to successful attachment of the gelatin layer. However, fibronectin recruitment due to the biocompatible coating showed insignificant differences in the biological response of the scaffolds [211]. *In vitro*, the cyclo(-RGDfK-) peptide promoted OB adhesion and proliferation with good biocompatibility. On the other hand, *in vivo,* reports revealed that modified porous tantalum stent could improve healing time in radial segmental bone defects in rabbits and can be considered for repairing big bone defects figure 21(b) [212]. Recently, these two approaches have been brought together where results suggest, the thermo-chemotherapy approach can only influence bone tissue attachment on the implant surface while peptide coatings prove more successful toward enhanced cell viability in the interior core of the implants [213]. Simvastatin/poloxamer 407 thermosensitive hydrogel employed to develop a compound system for AM porous Ti significantly increased vascularization was discovered in and around the porous Ti. The creation of new blood vessels and the volume of new bone have a strong relationship, figures 21(a), (c), (d), and (e) [214]. A porous metal compound demonstrates a viable way to improve bone ingrowth ability; multiple carriers loaded with various bioactive elements can be simply applied to generate diverse biological effects*. In vivo* investigations, bone and blood vessel growth may always be the most crucial proof.

## 5. Current challenges and future trends

The growing interest in the use of porous metals in orthopedic implants is indicated by the emergence of a vast number of scientific investigations studying the mechanical and *in vivo* behavior of porous metallic structures depending on the material properties, processing parameters, the morphology and distribution of the porosity, etc in the past decade. Due to their superior mechanical strength and resilience, metallic biomaterials are preferred for load-bearing implant applications. However, the main disadvantage of using metallic implants arise due to their higher stiffness when compared to bone. This stiffness mismatch affects the strength of the host bone tissue and thus causes aseptic loosening of the implant structure and reduces the implant’s lifetime. Also, most biocompatible metals are by nature bioinert and do not bond with the host tissue. Thus, it has been a common practice to use bone cement or porous coatings over metal implants to aid in implant fixation. However, bone cement is prone to brittle fracture, and the porous coatings tend to leach and corrode. Therefore, use of porous metallic structures as implant structures alleviates the issues of stress shielding by reducing the stiffness of the implant, and in the case of open pores, the tissue ingrowth into these pores ensures improved and more effective implant fixation. Despite these beneficial applications of porous metals in orthopedic implants, choosing an appropriate manufacturing method or processing parameters to get porous metals with optimum pore morphology remains challenging [15].

Most of the investigations concerning porous metals as implants are concerned with identifying an ideal fabrication method or processing parameters to better match the implant’s stiffness with the surrounding bone and improve the fixation of the implant. Another aspect of actively researching porous metallic implants relates to the inherent challenges of porous metals concerning a reduction in mechanical strength and lesser fatigue properties. Moreover, investigators aim to comprehend the different parameters of the fabrication of porous metallic implants and their effects on these implants’ mechanical and *in vivo* behavior. So far, it has been understood that designing and fabricating a porous implant structure is a multifactorial process that needs to take into consideration: the mechanical behavior of porous implant in comparison to the bone under several loading conditions, the corrosion-related issues, the ability for adherence with the bone tissue, pore morphology and distribution which affects the fatigue strength of the implant and in case of open-celled porosity the bone ingrowth [215].

Though much progress has been made in fabricating porous metallic structures with open or closed porosity using conventional or novel fabrication processes, several limitations persist. Firstly, for most fabrication processes, it is challenging to control the morphology, size, and distribution of the pores in porous implants, and in processes where this control is feasible, it is usually only possible over large areas. Secondly, most conventional fabrication processes use external chemical agents such as binders or foaming agents, etc, and these processing aids tend to leave residual impurities in the final structure that could harm the body. Finally, it is nearly impossible and highly uneconomical to manufacture porous metallic implants with designed porosity specific to any patient or site of the application using conventional manufacturing processes [79]. However, recent advances in AM technologies have provided several methods of fabricating metallic structures with designed porosity. Many commercially available orthopedic implants are customized to the patient and designed and fabricated using AM equipment such as EBM, LENS, etc. Also, AM technologies’ precision, resolution, and efficiency are expected to improve substantially, thereby introducing several opportunities to advance orthopedic implant technology further [216]. Recent research results also show that topology optimization can be used with metal AM to reduce stiffness without compromising the strength of porous metals [217, 218].

Understanding the benefits of porosity in metallic implant structures is crucial. It is commonly agreed that the next advancement would be in engineering these pore characteristics to achieve desired implant properties. It is thus implied that a fabrication process that could engineer the positioning, shape, size, interconnectivity, and distribution of pores is of most importance. In recent years, much research in the use of AM for fabricating porous metal implants has been undertaken owing to its capability to manufacture specifically designed or functionally graded porosities. However, it is still unclear what the optimum pore characteristics for an ideal porous metallic implant are and how to engineer them using various fabrication processes. Thus, future advancements in porous metallic implants might be possible by conducting investigations to obtain the optimum pore characteristics and processing parameters. Knowingly, fast-paced progress in AM of metallic structures implies vast scope for possibly having more control over the design of porosity specific to an application.
